# Transcriptome Analysis of *Scrippsiella trochoidea* CCMP 3099 Reveals Physiological Changes Related to Nitrate Depletion

**DOI:** 10.3389/fmicb.2016.00639

**Published:** 2016-05-09

**Authors:** Joshua T. Cooper, Geoffrey A. Sinclair, Boris Wawrik

**Affiliations:** ^1^Department of Microbiology and Plant Biology, University of OklahomaNorman, OK, USA; ^2^Department of Marine, Earth and Atmospheric Sciences, North Carolina State UniversityRaleigh, NC, USA

**Keywords:** transcriptome, *Scrippsiella trochoidea*, harmful algae, nutrient limitation, nitrogen, dinoflagellates, phytoplankton, algal physiology

## Abstract

Dinoflagellates are a major component of marine phytoplankton and many species are recognized for their ability to produce harmful algal blooms (HABs). *Scrippsiella trochoidea* is a non-toxic, marine dinoflagellate that can be found in both cold and tropic waters where it is known to produce “red tide” events. Little is known about the genomic makeup of *S. trochoidea* and a transcriptome study was conducted to shed light on the biochemical and physiological adaptations related to nutrient depletion. Cultures were grown under N and P limiting conditions and transcriptomes were generated via RNAseq technology. *De novo* assembly reconstructed 107,415 putative transcripts of which only 41% could be annotated. No significant transcriptomic response was observed in response to initial P depletion, however, a strong transcriptional response to N depletion was detected. Among the down-regulated pathways were those for glutamine/glutamate metabolism as well as urea and nitrate/nitrite transporters. Transcripts for ammonia transporters displayed both up- and down-regulation, perhaps related to a shift to higher affinity transporters. Genes for the utilization of DON compounds were up-regulated. These included transcripts for amino acids transporters, polyamine oxidase, and extracellular proteinase and peptidases. N depletion also triggered down regulation of transcripts related to the production of Photosystems I & II and related proteins. These data are consistent with a metabolic strategy that conserves N while maximizing sustained metabolism by emphasizing the relative contribution of organic N sources. Surprisingly, the transcriptome also contained transcripts potentially related to secondary metabolite production, including a homolog to the Short Isoform Saxitoxin gene (*sxtA*) from *Alexandrium fundyense*, which was significantly up-regulated under N-depletion. A total of 113 unique hits to Sxt genes, covering 17 of the 34 genes found in *C. raciborskii* were detected, indicating that *S. trochoidea* has previously unrecognized potential for the production of secondary metabolites with potential toxicity.

## Introduction

Harmful algal blooms (HABs) are a natural phenomenon (Hallegraeff, 1993; Granéli and Turner, 2006b), yet HAB frequencies and apparent ecological pervasiveness have increased within the last several decades. The formation of blooms occurs through the intersection of physical, chemical, and biological processes that are often specific to the HAB species (Paerl, 1988; Granéli and Turner, 2006a). Global prevalence and expansion of HABs appear, at least in some cases, to be linked to anthropogenic organic and inorganic nutrient loading into estuarine and coastal regions (Glibert et al., 2006; Anderson et al., 2008; Howarth, 2008). Agricultural runoff from the usage of inorganic nitrogen (nitrate and ammonium) has been shown to promote large phytoplankton algal blooms in the Gulf of California (Beman et al., 2005). Inorganic nitrogen has also been shown to promote many HABs as have organic sources of nitrogen such as urea, glutamine, glycine, and amino acids (Baden and Mende, 1979; Mulholland et al., 2002; Dyhrman and Anderson, 2003; Glibert and Legrand, 2006; Cochlan et al., 2008; Kudela et al., 2008). Similarly, HABs also have ways to incorporate organic forms of phosphate using secreted ectoenzymes (alkaline phosphatase) to hydrolyze the organic-P back to inorganic-P for uptake (Sakshaug et al., 1984; Dyhrman, 2005).

While some HAB occurrences appear to be strongly linked to nutrients, others show limited connections (Anderson et al., 2008). Instead, it appears that HAB dynamics exhibit complex relationships with biotic and abiotic factors. The strength of nutrient-HAB relationships are complicated by the variability in HAB adaptations to differing nutrient and light regimes (Smayda, 1997). In addition, many HAB species, such as some dinoflagellates, are capable of switching their dependence on strict photoautrophy to mixotrophy by feeding on bacteria, algae (Jeong et al., 2005a,b), or organic N and P from decaying fish killed by the bloom and zooplankton excretions (Vargo et al., 2008). In areas where nutrients limit growth, dinoflagellates may also migrate vertically to nutrient rich sediments to uptake dissolved N (Sinclair et al., 2006a,b; Sinclair and Kamykowski, 2008) thus alleviating nutrient stress. Given this, significant questions remain about the way in which many HAB species adapt to environmental variability at the molecular and cellular level, the way in which they conserve and utilize diverse dissolved organic and inorganic nutrients, and how requisite cellular mechanisms are controlled and can help us explain bloom persistence.

Dinoflagellates are a major component of marine phytoplankton and many species are recognized as toxin producing HABs (Smayda, 1997). Dinoflagellate bloom dynamics involve a complicated life cycle that includes stages of vegetative growth, sexual reproduction, and formation resting cysts (Xiao et al., 2003; Granéli and Turner, 2006a). Non-toxin producing dinoflagellates are less well studied than their toxic counterparts, but can frequently be as devastating to local fisheries via the formation of high-density, high-biomass blooms that result in hypoxia (Horner et al., 1997). *Scrippsiella trochoidea* is a non-toxic, marine dinoflagellate that can be found in both cold and tropical waters where it is known to produce “red tide” events. *Scrippsiella* blooms have been reported extensively from China (Qin et al., 1997; Wang et al., 2007; Zinssmeister et al., 2011), the coasts of Japan, Northern Europe, the Mediterranean, the Southern Atlantic of Namibia (Montresor et al., 1998; Gottschling et al., 2005; Spatharis et al., 2009), the Southern Gulf of Mexico (Licea et al., 2002), and the coastal United States (Zinssmeister et al., 2011). *Scrippsiella* blooms can become high in cell density and can lead to oxygen depletion resulting in fish kills (Hallegraeff, 1992).

The interplay between inorganic nutrients and *S. trochoidea* bloom formation appears complex. For example, a bloom of *S. trochoidea* in a semi-enclosed bay near Hong Kong maintained high cellular densities in the face of low inorganic nutrients (N, P, Si, metals), and bloom formation could not be stimulated via nutrient addition (Yin et al., 2008). Modeling instead suggests that diel vertical migration of *S. trochoidea*, and wind/tidal currents can cause convergences where cells are concentrated by physical forces even when waters are nutrient depleted (Lai and Yin, 2014). It has also been suggested that HABs may succeed in the wake of preceding nutrient depleting blooms of other phytoplankton species, allowing species adapted to low nutrient concentrations or feeding on bacteria or organic pools to thrive. *S. trochoidea* was traditionally considered to be strictly a photoautrophic dinoflagellate, however experimental feeding studies have shown *S. trochoidea* to be mixotrophic, ingesting organic matter or prey including other dinoflagellates, cryptophytes (Jeong et al., 2005b), diatoms (Du Yoo et al., 2009), and bacteria (Jeong et al., 2005a). In fact, most photoautrophic dinoflagellates are now thought to be capable of mixotrophy (Jeong et al., 2005b).

Here, we present a transcriptomic analysis of *S. trochoidea* CCMP 3099, using RNA-seq, designed to examine the effects of nitrogen limitation on gene expression. The purpose of the study was threefold. First, the study was part of the Marine Microbial Eukaryote Transcriptome Sequencing Project (MMETSP; Keeling et al., 2014), which in part aimed to characterize the diversity of protein coding genes in a broad diversity of marine algae. Dinoflagellates have some of the largest known genomes in nature as well as a high estimated genomic repeat content, which has made their genomes poor candidates for previous sequencing efforts. As a consequence, little is known about their complement of protein coding genes. Second, we investigated the transcriptional response triggered by N and P exhaustion, hypothesizing that regulation of gene expression would be in line with physiological adaptations related to nutrient limitation observed in other algal groups. Lastly, the genetic potential for toxin production was investigated by via an analysis of the transcriptome for the presence of transcripts encoding genes involved secondary metabolite production.

## Materials and methods

### Culture conditions

**Figure 1:**
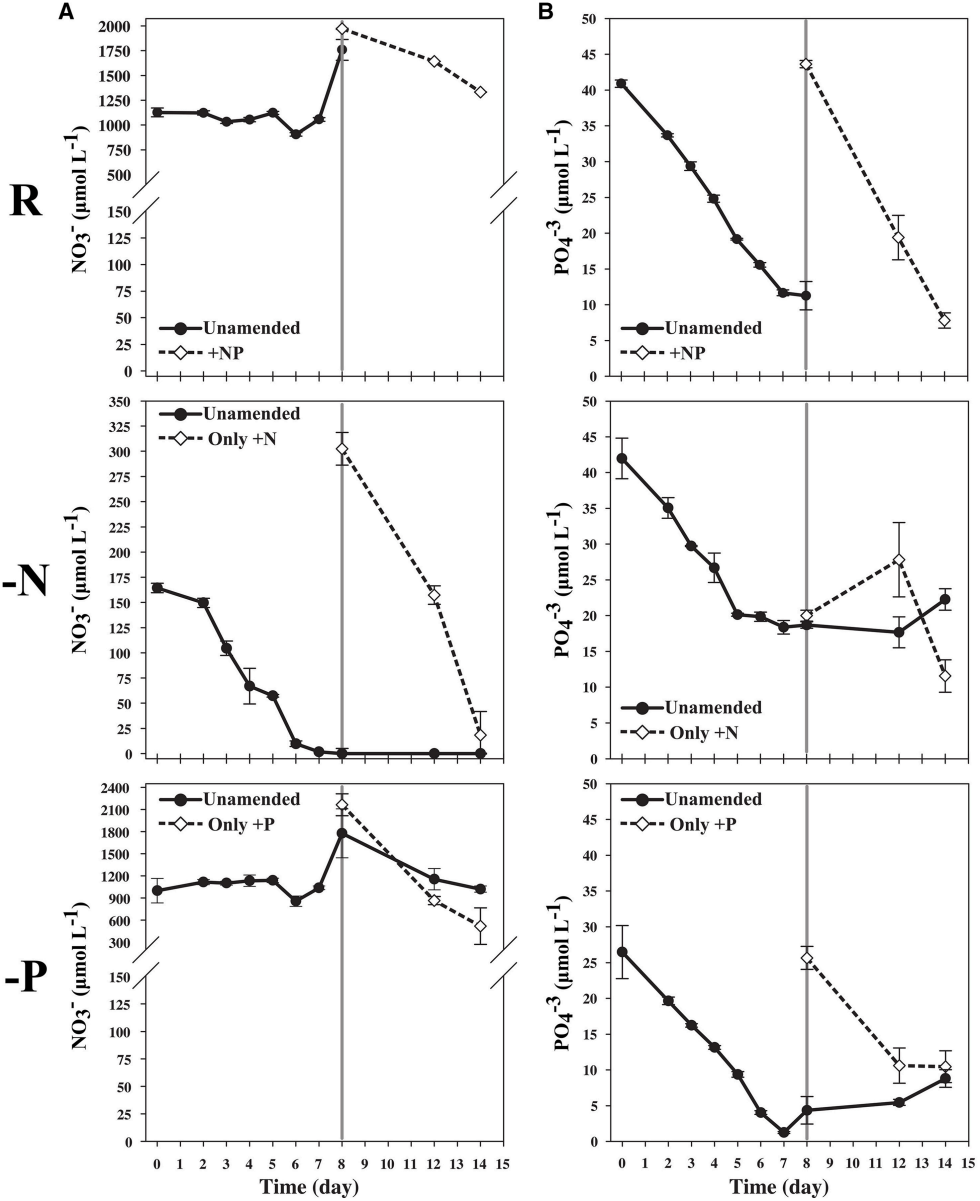
**(A)** Nitrate measurement illustrating low starting N concentration in the N-limited culture and depletion over time until day 7. Cultures, starved for 24 hours, were sampled on the 8th day. The remaining 300 mL were split into 150 mL cultures, and additional nitrate was added to one of the flasks originating from the N-limited treatment. **(B)** Phosphate measurements illustrating lower starting concentration of P in the P-limited culture, and depletion of P over time until day 7. The culture was starved 24 h, and sampled on the 8th day. The remaining culture was split into two equal volumes and phosphate was to one of the flasks originating from the P-limited treatment.

Non-axenic cultures of *S. trochoidea* CCMP 3099 were obtained from the National Center for Marine Algae and Microbiota (Provasoli-Guillard NCMA, Boothbay Harbor, ME). Cells were maintained in L1 (Guillard and Ryther, 1962; Guillard et al., 1973; Guillard and Hargraves, 1993) seawater media prepared from 0.45 μm filtered, autoclaved natural seawater. Seawater was obtained from the Gulf of Mexico at 33 ppt salinity, and was stored in the dark and aged for at least 3 months. Cultures were grown in a light incubator at 23–24°C and 30–40 μmol quanta·m^−2^s^−1^ light on a 12-h light:12-h dark cycle. Prior to the experiment, cells were grown to early stationary phase in 350 mL of L1 media and served as the inoculum for the nutrient trials at a 1:10 dilution. Concentrations of nitrate and nitrite were determined using the method of Miranda et al. (2001), which relies on the reduction of nitrate to nitrite with Vanadium(III). Nitrite in V-treated and untreated seater was then quantified via the addition of acidic Greiss regent and spectrophotometric detection at 535 nm. Phosphate concentrations were determined colorimetrically as per Grasshoff et al. (1983). Nutrient depletion rates were calculated as the difference in concentration between sequential time points divided by time (ΔC/t).The three treatments included the control (nutrient replete), nitrogen-limited, and phosphorus-limited and were run as static batches. To evaluate nutrient depletion responses in *S. trochoidea*, cultures were set up by modifying the ratio of available N to P. N:P ratios were modified so that cultures would grow into either exhaustion of N or P with roughly similar incubation times (Supplemental Table 1). Replete conditions were defined as the normal L1 media containing 880 μM NaNO_3_ and 36 μM NaH_2_PO_4_ (N:P ratio 24:1). Nitrogen-limited cells were started with an N:P ratio of 4:1 using 146 μM nitrate and 36 μM phosphate. Phosphorus-limited were started with a higher N:P ratio of 40:1, with nitrogen kept at replete levels (880 μM) and phosphate at 22 μM. Experimental cultures were grown in larger 1 L volumes of L1 medium in 2500 mL Pyrex Fernbach flasks (10 inch bottom diameter; < 1 inch medium) without shaking. All treatment flasks were gently mixed daily during the course of the experiment to eliminate possibility of either carbon limitation or patchy nutrient distribution within the Fernbach flask. Cell counts were generated daily to monitor growth by preservation with 1% formalin (v/v) and counting via light microscopy as well as using a Hemocytometer. Growth rates were calculated by using standard growth equations for exponential growth (Guillard, 1973). After sampling, cultures were transferred to new sterile culture flasks (150 ml volumes). The remainder of the N- and P-limited cultures were split, where one of the resulting sub-samples, respectively, was incubated under continued nutrient deplete conditions, while the other treatment was reconstituted to the original nitrate (N-deplete) or phosphate (P-deplete) concentration by nutrient addition (Figure 1).

### RNA isolation

Nutrient concentrations were monitored daily. Once nitrate or phosphorous dropped below detection limits (~1 μM for phosphate, and 0.1 μM for nitrate), cultures were allowed to continue growth for an additional 24 h to encourage complete depletion of the respective nutrients in culture medium and reduce the potential impact of residual nitrogen/phosphorus stored in vacuoles. All treatments were harvested during the mid-exponential growth phase 6 h into the 12-h light cycle. Cells were gently filtered in 25–50 mL aliquots onto 3 μm Durapore (Millipore) membrane filters at 5 PSI negative pressure to minimize cell lysis. The 3 μm filters have pore sizes sufficiently small enough to capture dinoflagellate cells, while allowing a large portion of bacterioplankton to pass through the filter. Filters were immediately transferred to 2-mL screw cap tube containing 750 μL RLT buffer (QIAGEN, Valencia CA) and ca. 50 mg of muffled glass beads (Biospek, Bartlesville, OK), frozen using liquid N_2_, and stored at −80°C until extraction. All samples for all treatments were taken within a 30 min window to reduce the potential effect of diel variations. For extraction, filters were thawed and cells were lysed by bead beating using a Mini-Bead Beater (BioSpec Products, Bartlesville, OK). Two rounds of beating were conducted at maximum speed for 2 min, placing tubes on ice for 2 min between steps. Cellular debris and filters were removed by centrifugation at 14,000 × g for 1 min. The supernatants were transferred to QIAshredder (Qiagen, CA, USA) columns to remove residual cellular debris. Total RNA was then extracted using the RNeasy Mini Kit (Qiagen, CA, USA) according to the manufacturers protocol. Genomic DNA bound to the column was removed using an on-column RNase-free DNase I digestion protocol (Qiagen, Valencia CA, USA) as recommended by the manufacturer.

### RNA library preparation and sequencing

RNA samples were quantified via a Qubit BR Single Stranded RNA Kit (Life Technologies, Grand Island, NY). The quality and integrity of RNA was assessed using the Aglient 210 Bioanalyzer. Library preparation and sequencing were performed by the National Center for Genomic Research (NCGR). Illumina TruSeq RNA sample preparation started from 2 μg of total RNA. The TruSeq protocol selects for mRNA by using a poly-T primer for bead capture and subsequent reverse transcription, limiting both ribosomal rRNA and prokaryotic mRNA contamination in the final sequence libraries. After bead capture and cDNA synthesis, libraries were generated by sheering fragments to an average 200–300 bp inserts size. Three separate cDNA libraries were sequenced with an Illumina HiSeq200 (Illumina, USA). The original sequence data can be obtained from the NCBI Sequence Read Archive under the accession numbers SRX551166, SRX551167, SRX551168 with MMETSP IDs of MMETSP0270, MMETSP0271, MMETSP0272 corresponding to the replete, nitrogen deplete, and phosphate deplete treatments.

### *De novo* transcriptome assembly

Several de Bruijn graph assemblers for RNAseq transcriptome reconstruction were assessed (data not shown) including Trinity, Velvet/Oases, and Abyss/Trans-Abyss (Birol et al., 2009; Simpson et al., 2009). Among the tested assembly algorithms, ABySS/Trans-ABySS performed best at reconstructing full-length domains of highly conserved sequences, as determined via repeated blastx (NCBI, blastx) queries. Raw Illumina reads were processed post-sequencing by NCGR to remove adapters. The trimmed read data provided by NCGR, still contained potential adapter artifacts and reads were therefore further processed in house to remove remaining residual adapters using Trimmomatic v.32 (Bolger et al., 2014) (Supplemental Table 2). Reads were then quality trimmed to remove low quality nucleotides with quality scores < 20. The *de novo* transcriptome of *S. trochoidea* was assembled by pooling data from all treatments together. Sequences were assembled using ABySS (v. 1.3.7; (Birol et al., 2009; Simpson et al., 2009) at 15 different *k-mer* settings ranging between 20 and 50 (stepwise increment of 2). The “erode” flag was set to zero and the number of pairs to consider a contig was set to 10 with scaffolding turned off as described elsewhere (Birol et al., 2009; Simpson et al., 2009). The multiple *k-mer* strategy was chosen as several studies have shown that small *k*-mer values can recover more short transcripts and are likely to be assembled while at larger *k*-mer values, fewer but longer transcripts are assembled (Surget-Groba and Montoya-Burgos, 2010). Large *k*-mers however enhance the possibility of closing gaps in shorter *k*-mer contigs, and thus a hybrid approach may recover complete fragments. Trans-ABySS version 1.4.8 (Robertson et al., 2010) was used to merge contigs from each single *k*-mer assembly into a final set of contigs. Trans-ABySS is a conservative merging algorithm, resulting in high redundancy. Contigs were therefore further collapsed and extended using the overlap consensus assembler CAP3 (Huang and Madan, 1999). Further CD-HIT-EST (v4.6; Li and Godzik, 2006) was applied as per the manual instructions for clustering expressed sequence tags so that smaller sequences with >90% identity to larger contig sequences would be collapsed. Previous studies of dinoflagellate transcripts suggest the possibility of many copies for individual genes (Bachvaroff et al., 2004; Hackett et al., 2004; Patron et al., 2005, 2006). Without a reference genome the approach taken here is likely a conservative underestimation of the true transcript diversity. To remove residual rRNA signal, blastn (Camacho et al., 2009) was used to compare reads to the SILVA Large Subunit (LSU build 115) and Small Subunit (SSU build 115) databases. Any read that matched a sequence in the SILVA databases with e-values < 1e^−50^ was removed.

### Transcriptome annotation

All contigs were searched using blastx (*e* < 1e-5) against the NCBI-NR, UniprotKB/Swiss_Prot, and UniprotKB/TREMBL databases. Annotation was conducted as outlined in (De Wit et al., 2012). Briefly, blastx hits are parsed for best hits, skipping hits for “hypothetical” or “unknown” proteins in favor of more descriptive terms. GO terms were assigned from Uniprot searches (De Wit et al., 2012). The pipeline also outputs Kyoto Encyclopedia of Gene and Genomes (KEGG) annotations. Krona was used to explore the taxonomy of hits to the NCBI-NR database and used to create Krona plots (Ondov et al., 2011). KEGG mapper (http://www.genome.jp/kegg/mapper.html) were used to examine KEGG biochemical pathway maps for critical pathways such as TCA cycle, Nitrogen Metabolism, Photosynthesis, and to examine the overall global transcriptome.

To further annotate sequences with poor blastx hit descriptions or lacked a database match, we choose to implement additional searches using RPS-BLAST against the CDD databases (COG, KOG, PRK, SMART). For this, assembled contigs were translated into potential amino acid sequences using ORFpredictor (Min et al., 2005). ORFpredictor uses a blastx like strategy to search for the best ORF, and also orders contigs in same reading framings. Previous transcriptome studies have reported that ORFpredictor performs well at finding the correct reading frame in dinoflagellate transcriptomes (Jaeckisch et al., 2011). Translated potential proteins were also annotated using HMMER3 hmmsearch (http://hmmer.janelia.org) against PfamA and PfamB (http://pfam.sanger.ac.uk), and Tigrfam (http://www.jcvi.org/cgi-bin/tigrfams/index.cgi) databases using the gathering thresholds for each model instead of an *e*-value threshold.

### Analysis for transcriptome completeness

A lack of genome data for *S. trochoidea* makes it difficult to assess whether transcriptomes have been sequenced at sufficient depth. In genome sequencing projects, genome completeness is often assessed by mapping data via the Core Eukaryotic Genes Mapping Approach (CEGMA) to estimate if core eukaryotic genes (CEGs) are present. As applied here, the core set of eukaryotic genes was based originally on the KOG (eukaryotic orthologous genes) classification, which has been refined to 458 CEG families (Parra et al., 2007). CEGMA was further developed to analyze those 248 ultra-conserved CEGs that are thought to be present in low copy numbers (Parra et al., 2009). The CEGMA output includes both complete ortholog as well as orthologs that are partial, and calculates a percent completeness for both partial and complete CEGs in addition the average number of orthologs per family. In addition to *S. trochoidea* transcriptome data, the CEGMA pipeline was also used to analyze previous public datasets downloaded from the NCBI Transcriptome Sequencing Archive, and genomes from JGI-DOE Genome portal that included dinoflagellates and other algae, which share close phylogenetic relationship to dinoflagellates.

### Read mapping and quantification of gene expression

The quality filtered, trimmed and rRNA free, paired-end fastq reads were mapped and aligned to the final transcriptome assembly using Bowtie2 (Langmead and Salzberg, 2012) and Samtools (Li et al., 2009). Bowtie2 was run with the “-sensitive”, “-no-mixed”, “no-discordant” parameters in “end-to-end” mode to only map reads that were paired properly and read counts were obtained using HTseq Count program (http://www.huber.embl.de/users/anders/HTSeq/) using “union” mode as the method to eliminate multi-mapping and transcripts that cover more than one contig. Examination of the transcriptome reveals fragmented genes and potential gene families. The resolution of the *de novo* transcriptome is limited to gene level and not isoform level quantification. Redundancy in *de novo* transcriptome assemblies may be artificial via the *k*-mer assembly strategy and could represent “real transcript variants/isoforms,” however the more conservative approach to collapse variants/isoforms was applied here. We note here that collapsing unigenes may lead to some loss of signal for differentially expressed paralogs. Raw counts were then used as input into the R package DESeq to detect differentially expressed genes, following the “without replicates” as outline in (Anders and Huber, 2010) and the DESeq manual. To estimate dispersion between samples without replicates, the method “blind,” and sharingMode set to “fit-only.” Only genes with adjusted *p*-values smaller than 0.1 representing false discovery rate were deemed differentially expressed (Benjamini and Hochberg, 1995).

### Functional enrichment

Functional enrichment of gene ontology were estimated using Fishers Exact test in topGO using the parent-child analysis to determine if genes identified as differential expressed were also enriched in biological processes, cellular components and molecular function (Alexa and Rahnenfuhrer, 2010). The adjusted *p*-values from the DESeq model of the replete vs. N-limited were used, as there were a number of genes identified as differentially expressed via the Benjamin Hochenberg adjusted *p*-value < 0.05. The node size was 10, and a *p*-value < 0.05 was applied to call GO categories as significantly enriched. Only those nodes that had *p*-value of < 0.01 are summarized.

### Analysis of secondary metabolite genes

Blast analysis (blastx and blastp; *e*-values < 1e-5) was used to compare *S. trochoidea* transcriptome sequences with the NCBI NR database, as well as sequence data sets from other algae, including dinoflagellates. For phylogenetic analysis (**Figure 5**), a subset of sequences was retrieved from the database, which included best database hits, as determined by blast, as well as more distantly related, representative sequences for comparison. Protein sequences were aligned using MAFFT version 7.157b (Katoh and Standley, 2013), and trimmed using trimAL with the “automated1” setting optimized for Maximum Likelihood reconstruction (Capella-Gutiérrez et al., 2009). The alignment was checked manually using SeaView 4 (Gouy et al., 2010) and all gaps and sites with sparse numbers of potential homologous amino acids were removed. The alignment was then further evaluated for the best amino acid substitution model using ProtTest 3 (Darriba et al., 2011), which suggested a model with LG+GAMMA. RAxML-SSE version 8 (Stamatakis, 2014) was used to reconstruct a maximum likelihood tree. The PROTGAMMALG setting in RAxML was used to search for the best tree in 100 searches, and subsequently calculated 100 bootstraps values. Additionally, the search method of (Hackett et al., 2013) was applied using protein sequences from *Cylindrospermopsis raciborskii* T3 saxitoxin biosynthesis cluster (Kellmann et al., 2008) to query to the un-translated *S. trochoidea* transcriptome using BLAST (tblastn, *e*-value < 1e-5) to identify additional Sxt genes in the transcriptome. In addition, secondary metabolite gene prediction was conducted via antiSMASH (Medema et al., 2011).

## Results

### Culture conditions

**Figure 2:**
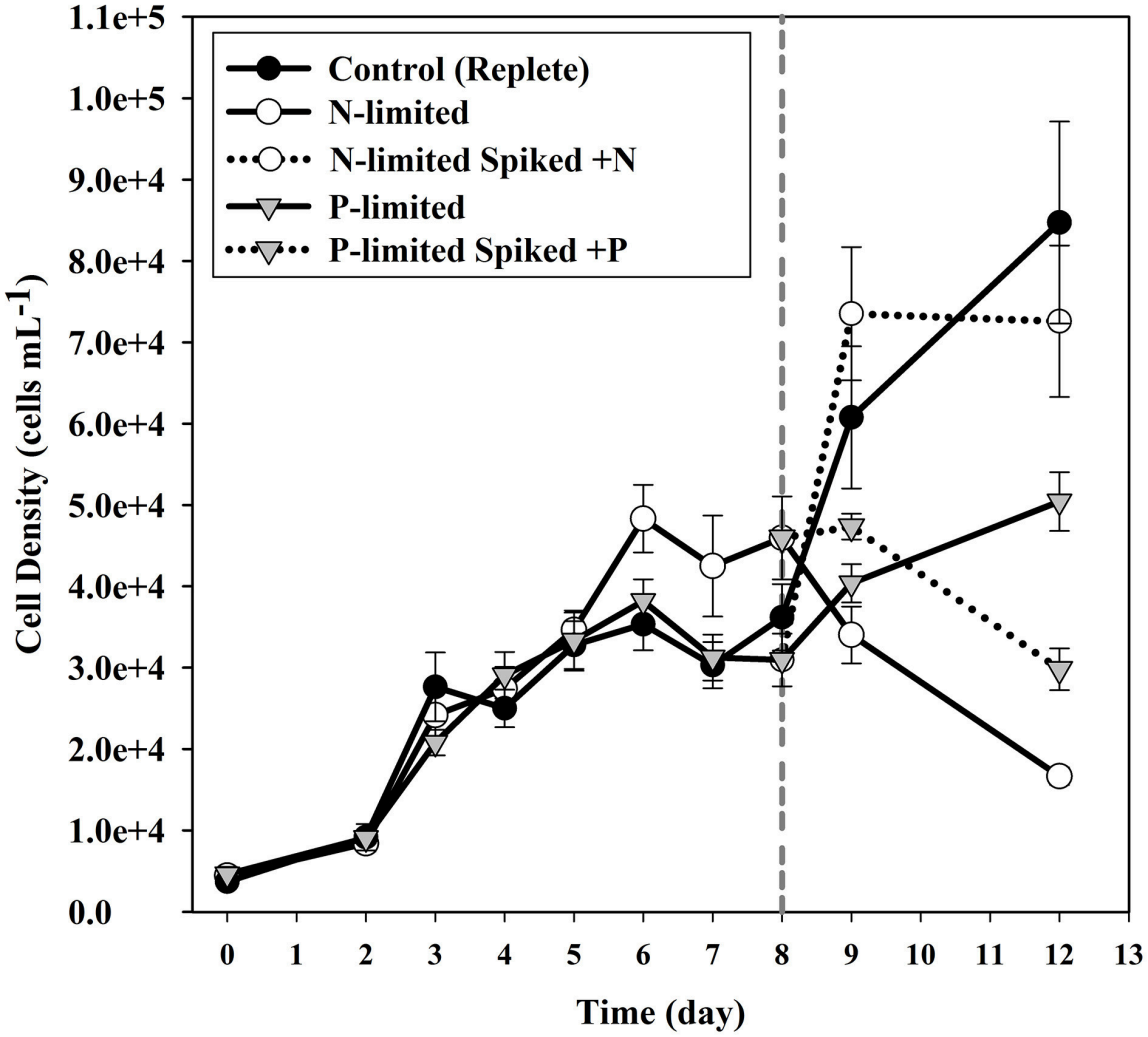
**Cell densities of cultures for each of the treatments**. Error bars indicate one standard deviation. Solid circles indicate the replete (control) cultures. Open circles are used to plot the N-limited cultures, while triangles are used to plot the P-limited cultures. Dotted lines indicate the sub-cultures that were spiked with nutrients after sampling.

During mid-log growth, cultures were depleting ca. 25 μM D^−1^ nitrate and 3.5 μM D^−1^ phosphate from the culture media (Figure 1). Given that the detection limits for the N and P assays utilized here (~1 μM), the assumption was made that residual nutrients were insufficient to maintain additional consumption 24 h after nutrient levels dropped below the limit of detection, and that a physiological response related to N and P depletion should be observed. Maximum growth rates for all three treatments were highest on day 3 with a maximum of 0.86 divisions per day for replete, 0.73 (d^−1^) for N-limited, and 0.91 (d^−1^) for P-limited treatments, respectively. Specific growth rates declined after day 3 in all cultures, as cell concentrations increased toward stationary phase and concomitant depletion of inorganic N and P. Immediately after sampling, the remaining culture volume (ca. 300 ml) was split into two equal volume sub-cultures, one of which was spiked with either 300 μM nitrate (for N-limited cultures) or 25 μM phosphate (for P-limited cultures) to demonstrate that growth would resume if limitation was relieved. In spiked flasks, cultures resumed rapid depletion for dissolved nutrients (Figure 1). The unspiked, N-limited culture continued to decline exhibiting net cell death, supporting the notion that the culture was indeed starved for N (Figure 2). Un-spiked P-limited cultures were able to maintain, albeit slow, growth suggesting that cells were not truly P-limited. In addition, measurable phosphate concentrations appeared to increase at and beyond the time of sampling in the P-limited culture (Paired *t*-test, *t* = −2.46, df = 2, *p* = 0.1331) (Figure 1). We note that cultures were transferred to a smaller culture flasks after sampling, which might have affected culture behavior. Cultures were, however, always maintained with high surface to volume ratios (large Fernbach flasks before sampling, and sideways incubated tissue culture flasks after sampling) to minimize impact of potential self-shading and light limitation.

### General transcriptome features

The assembled transcriptome of *S. trochoidea* consists of 201.9 million base pairs (Mbp) of sequence represented by 205,934 contigs (Supplemental Table 3). Redundancy reduction was used to collapse sequence isoforms and assembly errors using CAP3 and CD-HIT-EST, collapsing transcripts into 107,473 contigs with total length of 125 Mbp (Supplemental Table 3). Of these, 58 contigs produced significant hits to the Silva SSU and LSU rRNA databases and were removed from subsequent analysis, leaving a dataset of 107,415 complete or partial protein coding gene sequences.

**Figure 3:**
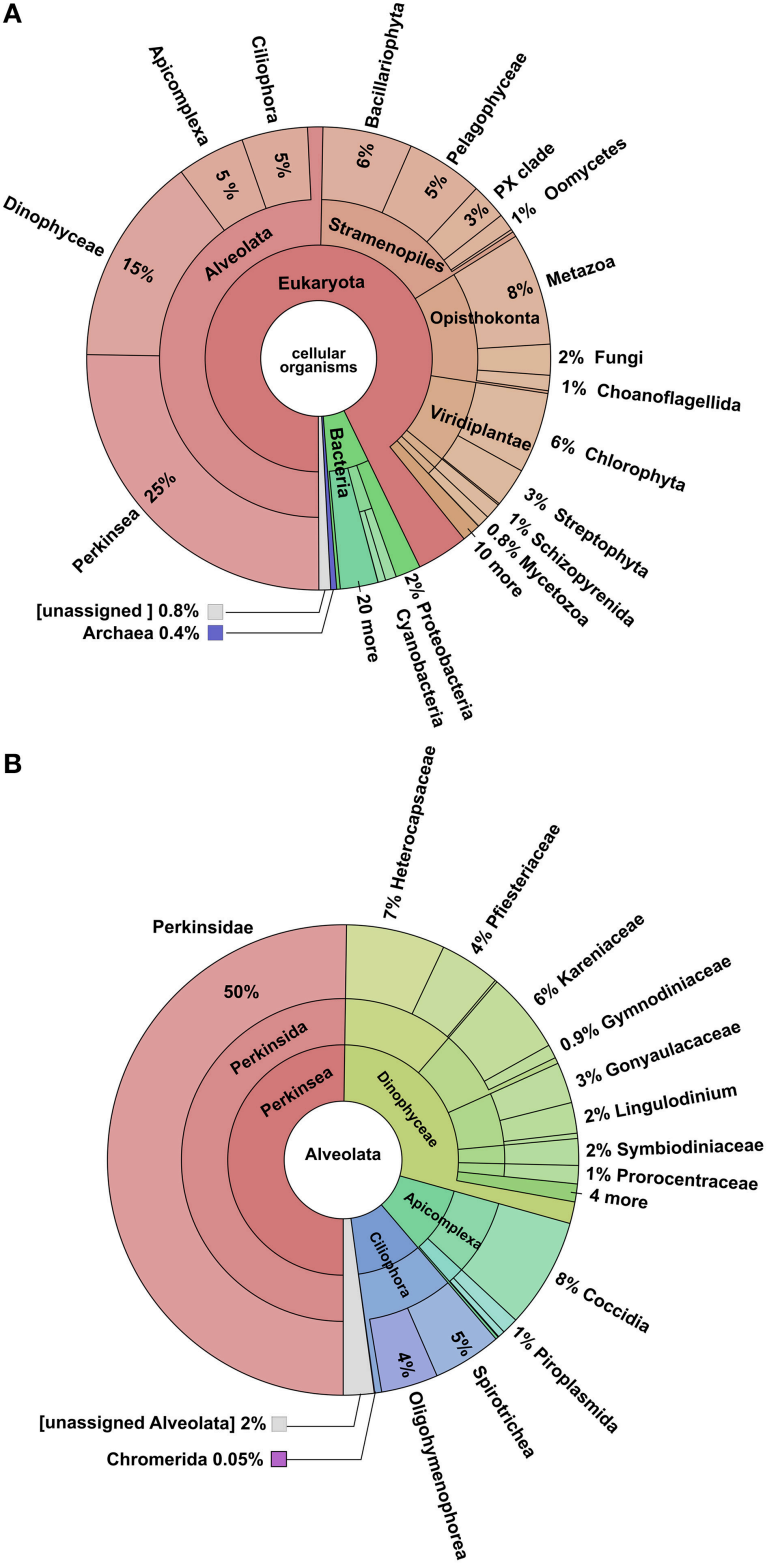
**Krona Plots of the blastx-hits**. **(A)** Distribution of all blast hits, showing that the majority was assigned to the Alveolata. **(B)** Hits within the Alveolata, showing that most reads were assigned to the *Perkinsus marinus*.

The blastx search of the NCBI-NR database yielded annotations for 41% of the total transcriptome. Of these, 93% were assigned to eukaryotes genes, 6% to Bacteria, and 0.4% to Archaea (Figure 3). Examination of hits within the Eukaryota showed that 50% could be assigned to Alveolata, 16% to the Stramenopiles, 11% to the Opisthokonta, 8% to Viridiplantae, while the remaining 15% belonged to a variety of less well represented clades. Most of the hit distribution within the largest group Alveolata yields most hits to *Perkinus marinus* (27%), Dinophyceae (16%), Apicomplexa (5%), and Ciliphora (5%). Blastx searches against Uniprot-SwissProt and Uniprot-TREMBL database yielded a combined 43,797 hits accounting for 40.1% of the total transcriptome as per De Wit et al. (2012). This generated 43,785 annotated transcripts. Of these, 16,148 transcripts could be assigned to enzymes in KEGG pathways, and 34,480 were assigned GO categories obtained from Uniprot-SwissProt. RPSblast with the conserved domain database (CDD) resulted in 31,725 contigs annotated with at least one CDD number, COG, Pfam, or SMART identification.

### Transcriptome completeness

The potential protein coding content of *S. trochoidea* was estimated using a non-linear model (Hou and Lin, 2009), which estimates the number of protein coding genes by integrating genomic information from sequenced eukaryotic organisms and cellular DNA content. Typically this number represents the haploid amount of DNA per cell, however, reports of genome size in *S. trochoidea* differ considerably, and *S. trochoidea* is known to make temporary cysts. Using values estimated by (Rizzo and Noodén, 1973) or (Shuter et al., 1983) of haploid cells at 17 or 34 pg DNA cell^−1^ gives a general estimate that *S. trochoidea* may contain between 58,464 and 66,579 protein-coding genes.

Alternatively, the completeness of ultra-conserved and conserved eukaryotic genes was estimated using the CEGMA pipeline, which uses a database of 248 single-copy, ultra-conserved eukaryotic genes originally based on the original eukaryotic orthologous groups (KOGs) as a measure of genome completeness (Parra et al., 2009). The CEGMA pipeline predicted 210 ultra-conserved CEG in the *S. trochoidea* transcriptome and an average of 2.74 orthologs per complete CEG. Of detected CEGs, 80.95% had more than one ortholog in the transcriptome. If CEGs with partial predictions are included, the number of ultra-CEGs increases to 220, suggesting that the transcriptome captures ca. 89% of the core genome in *S. trochoidea*, which is consistent with observations for other marine algae (Supplemental Table 5).

### Low phosphorus treatment

**Figure 4:**
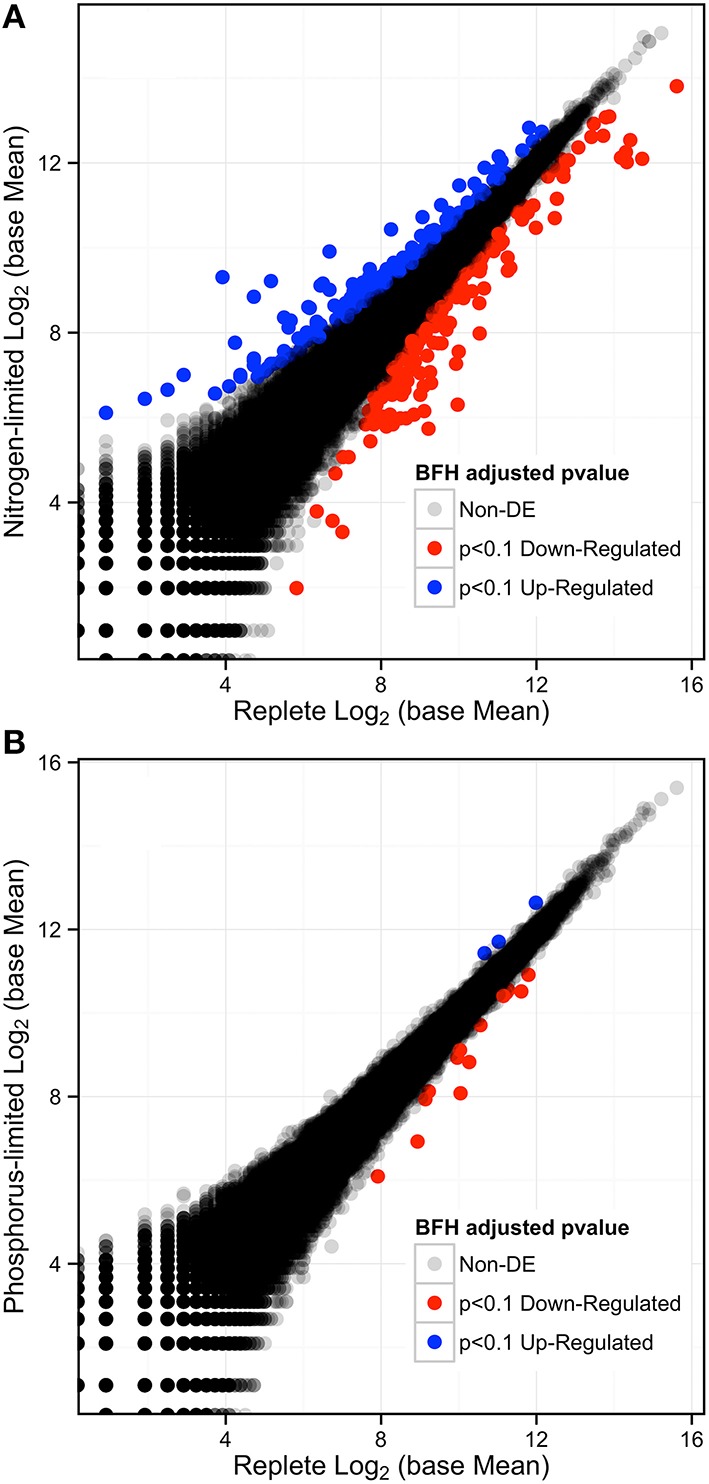
**(A)** Log-Log plot of expression levels comparing the replete to the N-depletion treatment showing the number of differentially expressed genes in red (down-regulated) and blue (up-regulated), and those that are no DE are in gray-black with p-adjusted to an FDR < 0.1. **(B)** Log-Log Plot of expression levels comparing the replete to the P-limited treatment.

The results from nutrient measurements and growth curves (Figures 1, 2) indicate that cultures did not reach P-limitation by the time of sampling even though phosphate concentrations had dropped below the limit of detection 24 h before RNA collection. The absence of limitation is supported by DESeq analysis of the respective transcriptome data, which indicated that only 17 transcripts (< 0.016%) were differentially expressed (*p*-adjusted < 0.1) (Figure 4B). Of these, 14 were down and three were up-regulated, while 12 were also significantly DE at an FDR = 0.05. Eleven transcripts had matches in the database. Among the downregulated transcripts were genes for sulfate transport, two homologs for fibrocystin-L, three genes for cell-wall binding proteins, and 3-dehydroquinate synthase. The only up-regulated transcript with a database match encodes the Photosystem Q(B) protein PsbA.

### Differentially expressed genes under N-Depletion

Analysis of the replete versus N-limited treatment using DESeq indicated that 382 transcripts were differentially expressed (DE) at a FDR = 0.1 (Figure 4A). Of these, 215 could be functionally annotated. Among the differentially expressed genes, 178 were also significantly DE at an FDR = 0.05. Of the 215 annotated DE genes, 107 were significantly down-regulated, and 108 were up-regulated in comparison to the replete control. DE transcripts were then clustered based on Biological Process and Molecular Function GO categories using topGO. Within each putative category, transcripts were ranked by Log2-fold change, with values < 0 representing down-regulation and values > 0 representing up-regulation relative to the control (Tables 1A, B). Analysis of the *S. trochoidea* transcriptome thereby demonstrates significant effects of nitrogen limitation on electron transport chain components, photosynthetic pathways, nitrogen, lipid, carbohydrate, and amino acid metabolism, as well as stress-related transcripts (Table 1). Using the adjusted *p*-values of the DESeq model as input to the GO enrichment test with topGO and an adjusted-*p*-value < 0.01 revealed similar patterns.

**Table 1A:** **Significantly enriched GO terms for Biological Process ***P*** < 0.01 with Positive Log2 fold change indicating up-regulation in response to N-depletion**.

**GO term**	**Description**	**Annotated**	**Significant**	**Expected**	***P*-value**
GO:0071705	Nitrogen compound transport	591	8	1.35	3.10E-04
GO:0044711	Single-organism biosynthetic process	2748	11	6.3	3.90E-04
GO:0019682	Glyceraldehyde-3-phosphate metabolic process	17	2	0.04	1.22E-03
GO:0008299	Isoprenoid biosynthetic process	120	3	0.27	1.41E-03
GO:0019288	Isopentenyl diphosphate biosynthetic process, methylerythritol 4-phosphate pathway	14	2	0.03	1.91E-03
GO:0046490	Isopentenyl diphosphate metabolic process	18	2	0.04	2.28E-03
GO:0044264	Cellular polysaccharide metabolic process	549	4	1.26	2.71E-03
GO:0007588	Excretion	35	2	0.08	3.49E-03
GO:0090407	Organophosphate biosynthetic process	831	6	1.9	3.67E-03
GO:0042886	Amide transport	107	3	0.25	7.21E-03
GO:0006793	Phosphorus metabolic process	3410	12	7.81	7.71E-03
GO:0051189	Prosthetic group metabolic process	14	1	0.03	1.05E-02
GO:0015833	Peptide transport	93	3	0.21	1.07E-02
GO:0009240	Isopentenyl diphosphate biosynthetic process	18	2	0.04	1.23E-02
GO:0006777	Mo-molybdopterin cofactor biosynthetic process	14	1	0.03	1.46E-02
GO:0040008	Regulation of growth	387	3	0.89	1.48E-02
GO:0006857	Oligopeptide transport	24	3	0.05	1.56E-02
GO:0005976	Polysaccharide metabolic process	695	4	1.59	1.60E-02
GO:0006081	Cellular aldehyde metabolic process	96	2	0.22	1.62E-02
GO:0006720	Isoprenoid metabolic process	168	3	0.38	1.68E-02
GO:0001558	Regulation of cell growth	141	2	0.32	1.86E-02
GO:0044765	Single-organism transport	4055	16	9.29	1.94E-02
GO:0008610	Lipid biosynthetic process	902	5	2.07	1.99E-02
GO:0000272	Polysaccharide catabolic process	402	3	0.92	2.02E-02
GO:0009726	Detection of endogenous stimulus	24	1	0.05	2.14E-02
GO:0009403	Toxin biosynthetic process	11	1	0.03	2.15E-02

See Supplemental Table 6 for complete list.

**Table 1B:** **Significantly enriched GO terms for Biological Process *P* < 0.01 with Negative Log2 fold change, indicating down-regulation in response to N depletion**.

**GO term**	**Description**	**Annotated**	**Significant**	**Expected**	**P-value**
GO:0022900	Electron transport chain	162	8	0.47	5.70E-08
GO:0015979	Photosynthesis	619	9	1.79	2.20E-05
GO:0009767	Photosynthetic electron transport chain	52	6	0.15	6.30E-05
GO:0018298	Protein-chromophore linkage	274	6	0.79	7.20E-05
GO:0006351	Transcription, DNA-templated	1394	8	4.04	1.10E-04
GO:0009636	Response to Toxic Substance	153	4	0.44	3.20E-04
GO:0044271	Cellular nitrogen compound biosynthetic process	2530	12	7.33	3.20E-04
GO:2000112	Regulation of Cellular Macromolecule Biosynthetic Process	1850	9	5.36	4.40E-04
GO:0006091	Generation of Precursor Metabolites And Energy	933	9	2.7	5.20E-04
GO:0055114	Oxidation-reduction process	902	10	2.61	5.60E-04
GO:0018130	Heterocycle biosynthetic process	2415	11	7	1.22E-03
GO:0019438	Aromatic compound biosynthetic process	2444	11	7.08	1.27E-03
GO:0009064	Glutamine family amino acid metabolic process	171	5	0.5	1.49E-03
GO:1901362	Organic cyclic compound biosynthetic process	2620	12	7.59	1.83E-03
GO:0032774	RNA biosynthetic process	1424	8	4.12	2.17E-03
GO:0010556	Regulation of Macromolecule Biosynthetic Process	1870	9	5.42	2.27E-03
GO:0006811	Ion transport	2297	17	6.65	2.32E-03
GO:0030509	BMP signaling pathway	20	2	0.06	2.47E-03
GO:0051340	Regulation of ligase activity	31	2	0.09	3.54E-03
GO:0015849	Organic acid transport	268	5	0.78	3.96E-03
GO:0071705	Nitrogen compound transport	591	7	1.71	4.08E-03
GO:0035335	Peptidyl-tyrosine dephosphorylation	17	2	0.05	4.90E-03
GO:0060255	Regulation of macromolecule metabolic process	2824	11	8.18	5.79E-03
GO:0000041	Transition metal ion transport	78	3	0.23	6.09E-03
GO:1903320	Regulation of protein modification by small protein conjugation or removal	139	3	0.4	6.75E-03
GO:0031396	Regulation of protein ubiquitination	125	3	0.36	7.52E-03
GO:0051252	Regulation of RNA metabolic process	1393	8	4.03	7.88E-03

See Supplemental Table 7 for complete list.

### Nitrogen metabolism under N-Depletion

**Figure 5:**
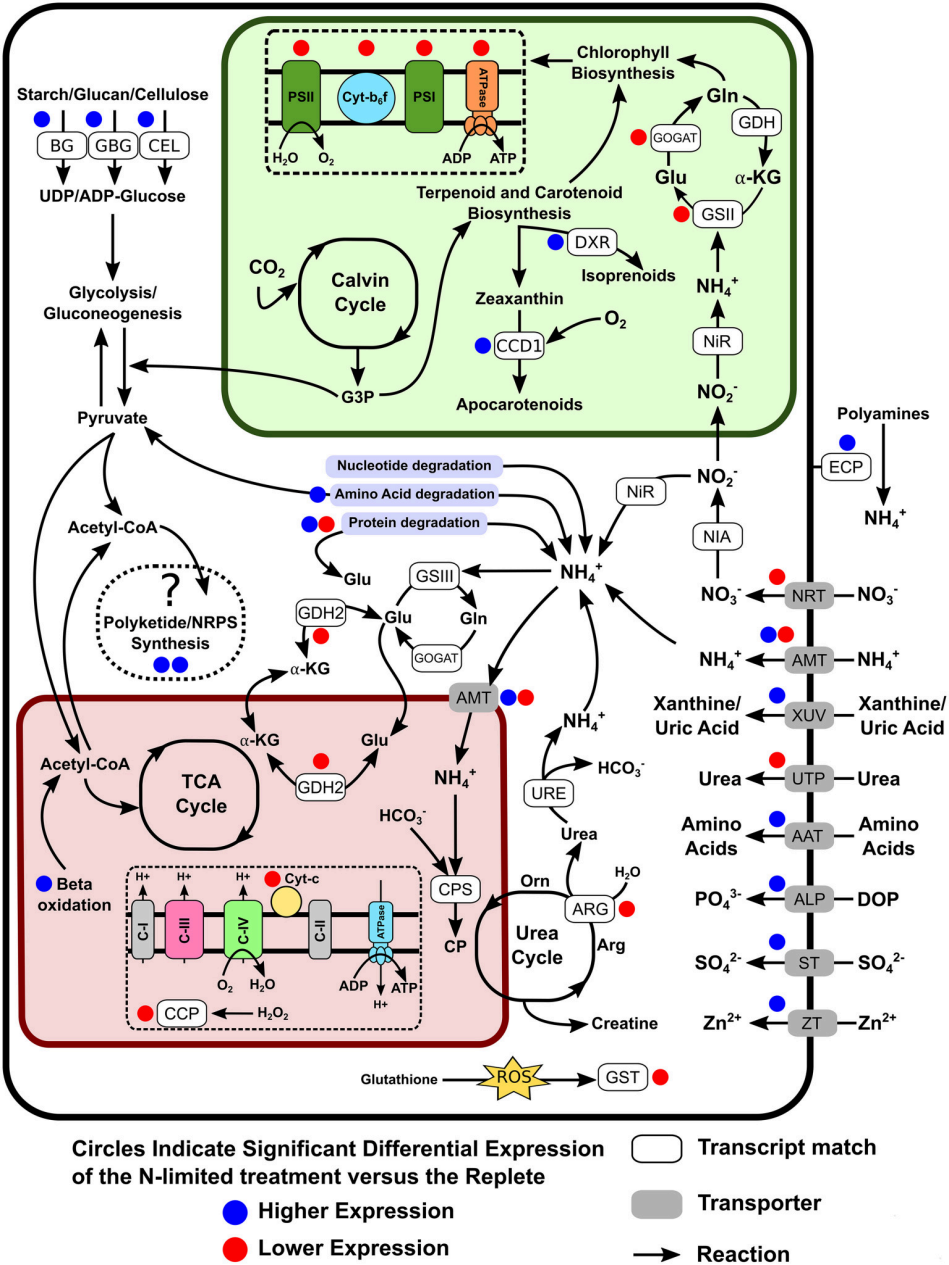
**Cellular overview of genes detected as differentially expressed and their potential roles in altering the metabolic pathways of *S. trochoidea* under N-limitation relative to replete conditions**. The glyoxylate cycle is not shown, connecting the mitochondrial and plastid pathways for clarity. Blue and Red circles indicate up- and down regulation in response to N-limitation respectively. AAT, Amino acid transporters; ALP, Alkaline phosphatase; AMT, Ammonium transporter; ARG, Arginase; BG, Beta-glucanase and 1,4-beta-D-glucan cellobiohydrolase; CCP, Cytochrome c peroxidase; CCD1, Carotenoid 9, 10(9′,10′)-cleavage dioxygenase 1; CEL, Cellulase/Endoglucanase 5 precursor; CP, Carbamoyl phosphate synthetase; DOP, Dissolved organic phosphate; DXR, 1-deoxy-D-xylulose 5-phosphate reductoisomerase; ECP, Extracellular peptidases/protease; GBG, Glucan 1,3-beta-glucosidase; GDH2, Glutamate dehydrogenase 2; GSII, Glutamine synthetase II; GSIII, Glutamine synthetase III; GST, Glutathione S-transferase; GOGAT, Glutamate synthetase; NIA, Nitrate reductase; NiR, Nitrite reductase; NRT, Nitrate/Nitrite transporter; STP, Sulfate transporter; URE, Urease; UTP, Urea transporter; XUV, Xanthine/Uric Acid transporter; ZT, Zinc transporter.

The transcriptome contained genes for the transport and reduction of nitrate, as well as homologs of glutamine synthetase and glutamate synthases (GS-GOGAT) corresponding to nuclear and plastid versions of these enzymes (Figure 5). Worth noting is that the detected nitrite reductase transcript was most closely related to a bacterial NAD(P)H nitrite reductase large and small subunits from the Flavobacteria *Formosa agariphila* KMM 3901 and *Joostella marina* DSM 19592, respectively. None of the primary nitrate assimilation genes were differentially expressed under N-limiting conditions. Several transcripts related to xanthine metabolisms (e.g., 10 contigs annotated as xanthine dehydrogenase), which have recently been proposed to be involved in N storage (Lin S. et al., 2015), were detected and were highly but were not differentially expressed. Several nitrogen cycling pathway genes were significantly down-regulated, including two glutamine amindotransferase-like proteins, a urea transporter, arginase, glutamate dehydrogenase, nitrate/nitrite transporter, and NADH dependent glutamate synthase. Genes most closely related to ammonia transporters displayed both up and down-regulated expression, with four transcripts experiencing significant down-regulation and two having significantly increased expression levels. Among the up-regulated transcripts related to N metabolism were genes for the uptake and degradation of proteins or amino acids such as uric-acid permease, xanthine-uracil permease, aspartyl aminopeptidase, polyamine oxidase, extracellular serine proteinase, and aliphatic amidase expression-regulating protein, and oligopeptide transporter 6, while others such as Leu/Ile/Val-binding proteins, and vacuolar amino acid transporter were down-regulated. Also among the DE genes were alkaline phosphatase, which was weakly up-regulated under N-depletion, as well as two contigs annotated as spore formation related proteins (spore coat protein A, subtilisin DY).

### Photosynthesis and cellular respiration under N-Depletion

In peridinin containing dinoflagellates such as *S. trochoidea*, the plastid genome has been reduced and mostly moved to the nuclear genome. Plastid DNA is therefore typically present as mini-circles that typically have only two functional genes (Hackett et al., 2004; Nisbet et al., 2004, 2008). The polyA-selection strategy was therefore able to retrieve multiple copies of all respective genes involved in the Calvin Cycle, and Photosynthesis (II and I), Cytochrome bc1 complex respiratory unit, and F-type ATPases. Under N-limiting conditions, genes representing transfer of electrons in Photosystems I & II (Photosystem II CP47, Photosystem I P700 A2, Photosystem I P700 A1, Photosystem II CP43, Photosystem II D2) along with addition members of the photosynthetic pathway (Photosystem Q, Cytochrome b6, Cytochrome b6-f complex subunit 4, Cytochrome b559, and Flavodoxin) were significantly down-regulated. This occurred in conjunction with the down-regulation of chloroplastic ATP synthase alpha and beta subunits and phototropin-2 related to chloroplast relocation. Genes involved in terpenoid biosynthesis, represented by 2 transcripts annotated as 1-deoxy-D-xylulose-5-phosphate reductoisomerase, as well as genes related to carotenoid biosynthesis were up-regulated under N-deplete conditions. Conversely, transcripts related to chlorophyll biosynthesis were expressed at similar levels in both treatments. In addition to photosynthesis related genes, a down-regulation in transcripts pertaining cellular respiration and detoxification of reactive oxygen species was observed (Cytochrome c peroxidase, sodium/calcium exchanger in the mitochondrial membrane, Cytochrome b, and Cytochrome c oxidase subunit 1).

### Carbohydrate and lipid metabolism under N-Depletion

KEGG analysis indicates that the *S. trochoidea* transcriptome contains all requisite genes for the reductive pentose phosphate cycle, glycolysis, gluconeogenesis, glyoxylate cycle, galactose degradation, fatty acid biosynthesis (initiation, elongation), beta-oxidation, and the tricarboxylic acid cycle (TCA). Complete pathways for lipid metabolism of several compounds were detected, including triacylglycerol biosynthesis, acylglycerol degradation, ceramide, and sphingosine biosynthesis, as well as sphingosine degradation. Also present are a complete set of transcripts necessary for the biosynthesis of glucose to UDP-glucose and galactose to UDP-galatactose. The primary storage product in dinoflagellates is starch (Seo and Fritz, 2002; Lee, 2008; Dagenais Bellefeuille et al., 2014), and it has been shown that starch biosynthesis may begin with UDP-glucose in dinoflagellates (Deschamps et al., 2008). The transcriptome also suggests terpenoid biosynthesis of C5 isoprenoids via the non-mevalonate pathway. Transcripts related to carbohydrate catabolism of cellulose appear to be significantly up-regulated under N-depletion. These include beta-glucanase, 1,4-beta-D-glucan cellobiohydrolase B, 1,4-beta-D-glucan cellobiohydrolase B, glucan 1,3-beta-glucosidase, and Endoglucanase-5.

### Secondary metabolite genes under N-Depletion

**Figure 6:**
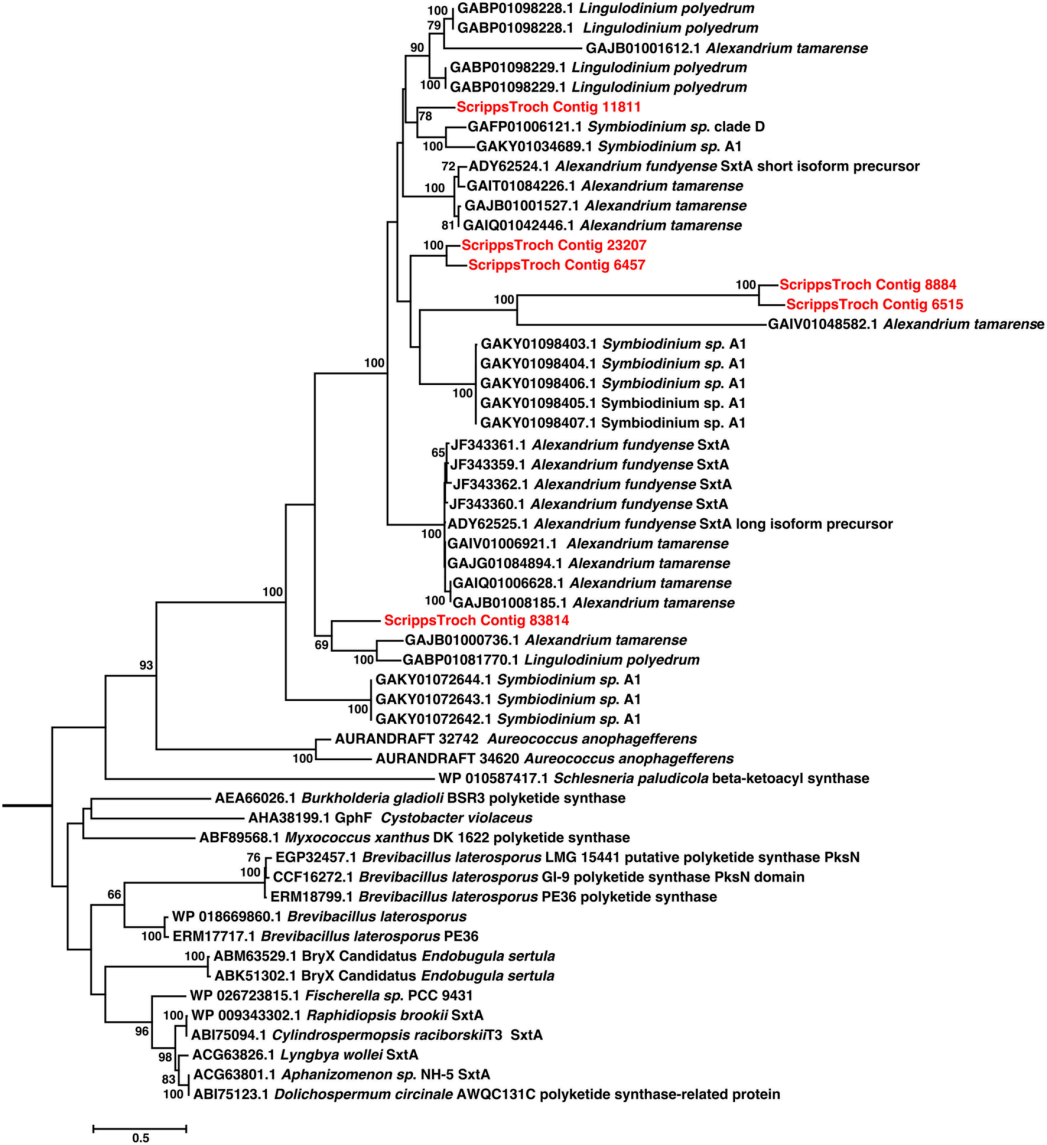
**Phylogenetic relationships among Saxitoxin *sxtA* transcripts from *S. trochoidea* and close blastp hits to NCBI-NR**. Twenty Maximum likelihood searches and 500 Rapid Bootstraps were preformed using RAxML. *sxtA* hits from *S. trochoidea* form a clade with others from dinoflagellates. These are related to the sister clade of *sxtA* from cyanobacteria which are known to produce toxins.

**Figure 7:**
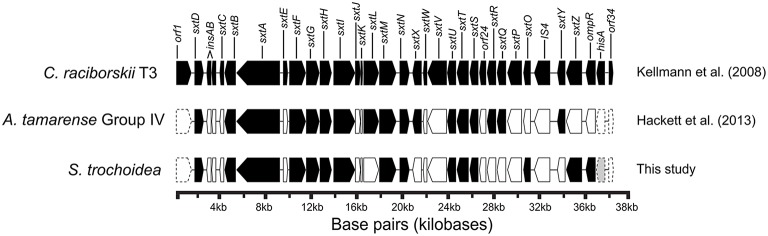
**Gene arrangement of Saxitoxin gene cluster in *Cylindrospermopsis raciborskii* with homologs of *Alexandrium tamarense* and *Scrippsiella trochoidea* shaded to demonstrate shared gene similarity in both toxic and non-toxic dinoflagellates and the known cyanobacterial saxitoxin pathway (Adapted from Kellmann et al., 2008), and *sxt* gene hits of *Alexandrium tamarense* plotted from Hackett et al. (2013) in addition *sxt* gene hits from this study**.

Blast analysis of the *S. trochoidea* transcriptome revealed several transcripts potentially related to secondary metabolite production, including a hit to the Short Isoform Saxitoxin gene (*sxtA*) from *Alexandrium fundyense*, which was up-regulated under N-depletion, as well as several additional polyketide synthases. Blast analysis (blastx and blastp; *e*-values < 1e-5) was used to compare *S. trochoidea* transcriptome sequences with the NCBI NR database, as well as sequence data sets from other algae, including dinoflagellates. In all, seven transcripts were annotated as “*sxtA* short isoform” in this manner. Five of these were longer than 200 amino acids and were phylogenetically analyzed (Figure 6). The discovery of a homolog to the *sxtA* genes in *S. trochoidea* prompted a search for the remainder of the saxitoxin biosynthetic cluster as described for *C. raciborskii* described by (Kellmann et al., 2008). All 34 annotated Sxt peptides from *C. raciborskii* were therefore tblastn (*e* < 1e-05) searched against the *S. trochoidea* transcripts. A total of 136 contigs exhibited homology to the saxitoxin biosynthesis cluster (Table 2). Of these, 113 were unique hits to Sxt genes, covering 17 of the 34 genes found in *C. raciborskii, indicating that S. trochoidea* appears to posses homologs to at least half of of the biosynthesis pathway involved in the synthesis of saxitoxin (Figure 7). *S. trochoidea* also appears to show similar presence of 12 Sxt homologs also found in *Alexandrium tamarense* Group IV (Hackett et al., 2013).

**Table 2:** **Transcriptome gene hits to the Saxitoxin biosynthesis gene cluster in *Cylindrospermopsis raciborskii* T3**.

**Gene name in *Cylindrospermopsis raciborskii* T3**	**Name**	**Count**	**Average *E*-value**	**Average AA**	
				**%Similarity**	**HSP Length**
SxtA polyketide synthase-related protein	SxtA	10	1.00E-06	48%	433.7
SxtB cytidine deaminase	SxtB	1	7.00E-36	55%	233.0
SxtD Sterol desaturase	SxtD	1	7.00E-08	46%	155.0
SxtF sodium-driven multidrug and toxic compound extrusion protein	SxtF	1	5.00E-04	41%	358.0
SxtG amidinotransferase	SxtG	4	5.00E-13	50%	291.8
SxtH phenylpropionate dioxygenase	SxtH	16	1.72E-06	44%	186.1
SxtI NodU/CmcH-related carbamoyltransferase	SxtI	3	3.33E-07	50%	327.7
SxtN sulfotransferase	SxtN	1	6.00E-05	39%	285.0
SxtO adenylylsulfate kinase	SxtO	2	5.10E-45	66%	177.0
SxtS phytanoyl-CoA dioxygenase	SxtS	2	3.60E-04	43%	181.0
SxtT phenylpropionate dioxygenase	SxtT	14	2.79E-06	45%	187.0
SxtU short-chain alcohol dehydrogenase	SxtU	53	2.14E-05	48%	146.9
SxtW ferredoxin	SxtW	5	1.85E-04	51%	64.8
SxtZ histidine kinase	SxtZ	11	3.69E-07	44%	278.7
HisA-related protein	HisA	1	5.00E-05	51%	73.0
Transcriptional regulator OmpR family	ompR	10	6.92E-07	59%	128.3
Unknown	–	1	4.00E-22	69%	94.0
Total Sxt gene pathway hits		136			
Number of Unique *S. trochoidea* contigs		112			

Approximately 50% of the biosynthesis cluster had significant hits to S. trochoidea (17 of the 34).

Phylogenetic analysis revealed that *S. trochoidea sxtA-like* transcripts formed a well-supported clade with sequences from other dinoflagellates (Figure 6), and that sequences originating *Aureococcus anophagefferens* appearing basal to dinoflagellate sequences. Cyanobacterial *sxtA* sequences clustered separately from dinoflagellates as has been shown previously (Hackett et al., 2013). Analysis of the Pfam annotation of putative *S. trochoidea* proteins and related sequences reveals the presence of a phosphopantetheine attachment site (PF00550). The *sxtA* short isoform of *A. fundyense* contains only a phosphopantetheine attachment site, while the “long” isoform also contain aminotransferase class I and II domains which are absent in the transcript detected in *S. trochoidea*.

In all, 235 putative polyketide synthase hits were detected in *S. trochoidea* when compared to *Emiliania huxleyi, Cryptosporidium parvum, Ostreococcus lucimarinus, Ostreococcus tauri, Chlamydomonas reinhardtii*, and dinoflagellate annotated transcripts in NCBI. Secondary metabolite prediction by antiSMASH showed these putative PKS hits grouped into 10 clusters. Three clusters represented Type I Polyketide synthases, three clusters of non-ribosomal peptide synthases (NRPS), and one cluster for hybrid non-ribosomal polyketide/polyketide synthase. Many of the contigs appears to have single beta-ketoacyl synthase (PKS_KS) domains, single acyl carrier protein domains (ACP), and polyketide synthase acyl transferase domains (PKS_AT). Contig 91643 was annotated as a Type I Polyketide synthase and was characterized by acyl carrier protein (ACP), repeating beta-keto-acyl synthase (KS), followed by dehydratases (PKS_DH), ketoreductase (PKS_KR), and ending with enoylreductsae (PKS_ER) and thioesterase (PKS_TE). Contig 84599 also had several repeating domains of KS, KR, DH, and ended with PKS_ER and PKS TE. The remaining contigs were highly fragmented and contained single or multiple domains. Regardless, these data point to significant potential to encode secondary metabolites in the *S. trochoidea* transcriptomes described here.

## Discussion

### General transcriptome features and completeness

Gene expression in eukaryotes is subject to a range of control mechanisms such as the level of transcription, nuclear export, translation, and posttranslational modification. It has been noted that perhaps a scarcity of transcriptional regulation exists in dinoflagellates (Lin, 2011). Similarly, proteomic analysis of *T. pseudonana* cultures have demonstrated a reduction of proteins related to nitrate reduction under N starvation even though they did not appear differentially expressed (Hockin et al., 2012), and post-transcriptional regulation in the presence of nitrate has been observed in *Cylindrotheca fusiformis* (Poulsen and Kröger, 2005). The present study may therefore underestimate the degree to which gene expression is modulated in *S. trochoidea*.

Though large, the size of *S. trochoidea*'s transcriptome is consistent with studies, which have reported ~49–118 K transcripts for dinoflagellates (Bayer et al., 2012; Beauchemin et al., 2012; Zhang et al., 2014). Similarly, ~42,000 predicted coding genes have been observed from sequencing approximately half the nuclear genome of *Symbiodinium minutum* (Shoguchi et al., 2013), which is thought to represent a basal clade of dinoflagellates and contain a smaller genome than other dinoflagellate lineages, while ~36,000 coding genes were reported for an assembly of ~80% of the genome of *Symbodinium kawagutii* (Lin S. et al., 2015). Analysis of ultra-conserved genes further indicates genome completeness of 85–89%, suggesting that transcriptomes described here are likely a good representation of the potential protein coding potential of *S. trochoidea.* Although selection of polyadenylated transcripts and size filtration were used to capture primarily eukaryotic poly-adenylated mRNA, some prokaryotic ribosomal RNAs (rRNA) were detected in the transcriptome assembly. However, these represented < 0.02% of reads. Assuming that the bacterial RNA pool was >95% ribosomal, it can be estimated that < 0.05% of the transcriptomes analyzed here were of bacterial origin and that bacterial contamination is of minor importance. Nevertheless, the best blast hits of *S. trochoidea*'s predicted proteins are often related to bacteria and archaeal homologs. Closer inspection shows that these hits typically exhibit low identity (~20–48%) amino acid similarity (blastx) and therefore likely arise as a consequence of the paucity of available protistan (eukaryotic) genomic data. Dinoflagellates also have unique and complex evolutionary histories of endosymbiosis, horizontal gene transfer, and vertical inheritance from both Bacteria and other Eukarya (Beauchemin et al., 2012; Wisecaver et al., 2013).

Approximately half of the transcriptome could be assigned annotations via comparison with NCBI-NR, and Uniprot-Swissprot/TREMBL databases. A lower percentage of assignments could be made via KEGG and KOG match annotations accounting for only ~28% and 22% of transcripts, respectively (Supplemental Table 4), which is consistent with observations for other dinoflagellates (Jaeckisch et al., 2011; Bayer et al., 2012). The paucity of available protistian genome sequences makes annotating these unusual eukaryotes challenging, because sequence similarities to the databases records are frequently < 40%, and it remains difficult to determine copy numbers or to identify isoforms. *S. trochoidea's* transcriptome contains many examples of highly similar transcripts, but it remains unclear to what degree these are artifacts of the multi-*k*-mer assembly strategy. Dinoflagellates have been shown to contain multiple distinct gene copies using EST and Sanger sequencing (Okamoto et al., 2001; Bachvaroff and Place, 2008), which do not suffer requisite assembly issues. The number of contigs reported here, therefore, is likely to be an over-estimate of the true diversity of proteins produced by *S. trochoidea*. Conversely, post-transcriptional processing and splicing may lead to assembled transcript diversity not reflected in the genome sequence. For example, homology among gene families and significant mRNA editing in dinoflagellates (Zhang and Lin, 2008) may produce unique *k*-mers that could produce spurious contigs. Also, 5′- spliced leader sequences produced by dinoflagellates (Zhang et al., 2007) may lead to artifacts, given that the de Bruin-graph depended assembly strategy is limited by *k*-mer diversity within reads, which may be narrow in the 5′-region of transcripts. Lastly, while it has been traditionally assumed that dinoflagellates genes have few introns, recent reports suggest that introns may be more wide spread than previously thought (Bachvaroff and Place, 2008; Orr et al., 2013; Shoguchi et al., 2013; Lin X. et al., 2015).

### Phosphorus metabolism

A significant response to low P conditions was not observed in the sequenced transcriptome. Only 17 genes were differentially expressed with respect to the replete treatment, and these were not characteristic of classical phosphorus limitation known from other dinoflagellates (Dyhrman and Palenik, 1999; Morey et al., 2011). For example, alkaline phosphatase is an ectoenzyme that hydrolyzes organic phosphates into dissolved phosphate for subsequent uptake and is commonly used as an indicator of phosphate stress in dinoflagellates (Dyhrman and Palenik, 1999). In *S. trochoidea* alkaline phosphatase activity has been reported to be low in nutrient replete and high in P-deficient cells (Sakshaug et al., 1984). The transcriptome contains several homologs to alkaline phosphatase, but these were not among the differentially expressed genes. Given this, all subsequent discussion focuses to the N-depletion experiment.

### Nitrogen metabolism

*S. trochoidea* displayed classic N starvation characteristics common in photosynthetic eukaryotes, including chlorosis (yellowing of the culture) and down-regulation of photosynthetic electron transport (Turpin, 1991; Morey et al., 2011). In addition, *S. trochoidea* experienced an up-regulation of genes related to amino acid catabolism and transport, consistent with increased processing and recycling of organic N compounds and remodeling of the internal metabolic pathways to compensate for the lack of external N. The observed connection between photosynthesis and N metabolism has long been recognized in green algae, diatoms, terrestrial plants (Turpin et al., 1988; Turpin, 1991), and the dinoflagellate *Alexandrium minutum* (Yang et al., 2010), where photo-acclimation through a reduction in photosystem reaction centers is linked to cells entering stationary-phase. Experiments described here were not designed to test exponential versus stationary-phase effects, and it is therefore difficult to separate the potential impact that growth phase responses may have played. However, both N-replete as well as N-spiked cultures continued to grow past the sampling time, while N-depleted cultures stagnated, indicating that *S. trochoidea* was likely not experiencing resource limitations other than those that were controlled for at the time of sampling.

Glutamine synthetase and glutamate synthase were among the significantly up-regulated transcripts, indicating increased investment in cellular ammonium assimilation potential. In contrast, the detected transcripts for nitrate and nitrite transporters as well as nitrate and nitrite reductases were not differentially expressed. This is in contrast to observations from diatoms, where nitrate and nitrite reductases are frequently up-regulated during N starvation. For example, in the diatom *Phaeodactylum tricornutum* nitrate assimilation genes, including nitrate and nitrite reductase and ammonium transporters, have been found up-regulated under N starvation (Maheswari et al., 2010). It should be noted that internal stores of nitrate/nitrite were not assessed in experiments describe here and it is possible that these were not entirely depleted by the time of sampling. Phytoplankton replete with nutrients are known to luxury consume and store in excess nutrients in vacuoles (Reynolds, 2006; Lin et al., 2016). In dinoflagellates this is often coupled to behavioral adaptation, such as vertical migration into nutrient rich sediments, where N uptake can support growth in a more nutrient deplete photic zone (Sinclair et al., 2006b, 2009). However, it has been noted that *S. trochoidea* does not appear to luxury consume (Flynn et al., 1996), and it is therefore more likely that cells adapted to nitrate depletion by adjusting overall cellular N processing and potentially targeting organic N substrates (see discussion below).

Several ammonium transporters were detected and observed to be both up- and down-regulated in *S. trochoidea's* transcriptome. Typically, ammonium transporter activity is highly expressed under nitrogen limitation. For example, ammonia and nitrate transporters were highly expressed under nitrogen limitation in *P. tricornutum*, and also in nitrogen-deficient media in the haptophyte *Isochrysis galbana* (Kang et al., 2007). Highest ammonium transporter mRNA was detected in nitrogen-starved *Cylindrothea fusiformis* (Hildebrand, 2005). In *S. trochoidea*, differential transcription of multiple ammonium transporters may reflect the utilization of ammonium transporters with differing affinity as is the case in *C. fusiformis* (Hildebrand, 2005). Upregulation of ammonium transporters has also been reported in prior studies. For example, when *Karenia brevis* (Wilson clone) was nitrate limited, gene expression of type III glutamine synthetases, nitrate/nitrite transporters, and ammonium transporters were all significantly up-regulated (Morey et al., 2011). Similarly, transcriptome analysis of *A. fundyense* indicated that this organism can utilize ammonium, nitrate, nitrite, urea and potentially cyanate, and when cells were N-limited significant up-regulation of nitrogen transporters, nitrite reductase, and glutamine synthetase was observed (Zhuang et al., 2015). Moustafa et al. have observed differential gene expression in *Alexandrium tamarense* in response to N and P limitation, as well as the presence or absence of bacteria, and have noted that the presence of bacteria was perhaps the primary driver associated with these changes (Moustafa et al., 2010). Overall, however, these data point to the interpretation that dinoflagellates are capable of incorporating many forms of dissolved inorganic and organic nitrogen sources to satisfy their N demand.

Transcriptome data presented here suggest that *S. trochoidea* may have remodeled cellular metabolism to make greater use of dissolved organic nitrogen (DON) compounds, pointing to increased importance of organic-N utilization under N-limiting conditions. This notion is supported by the observation that one of the most highly differentially expressed transcripts was a proton-coupled amino acid transporter with high sequence identity to *E. huxleyi* (Supplemental Table 8). In marine systems, DON production by microbes can be quite high (Bronk et al., 1994), and this is often mirrored by equally high uptake rates of recently produced DON (Bronk and Glibert, 1993). These observations indicate a tight coupling between the production and consumption of a pool of labile DON that represents an important source of N to the phytoplankton and bacteria in the environment (Sipler and Bronk, 2015). The labile DON pool is composed of metabolites such as amino acids and nucleosides, which are easily integrated into cellular metabolism. The uptake of DON compounds would be facilitated by the observed remodeling of the cellular pathways to compensate for nitrogen flow by recycling amino acids and unconventional nitrogen storage products such as uracil/xanthine. For example, dinoflagellates may store N in the form of uric acid crystals, which could later be catabolized when the cell are stressed (Dagenais-Bellefeuille and Morse, 2013). Elevated expression of a putative xanthine/uracil/vitamin C permease is consistent with transcriptomes reported for *S. trochoidea* (this study), *A. anophagefferens*, and *T. pseudonana* (Mock et al., 2008; Wurch et al., 2011). While the functional diversity of xanthine permease-like enzymes is broad, they potentially aid in uptake of purines from the environment, and may serve as superior sources of nitrogen when limited. This is supported by a study of *A. anophagefferens* in which xanthine permease was high up-regulated when cells were N limited in both culture and field conditions (Wurch et al., 2014). Overall, it therefore appears that *S. trochoidea* may be well poised to benefit from the rapidly cycling DON pool in lieu of available DIN sources, at least when nitrate is initially depleted.

### Carbon metabolism

Nitrogen stoichiometrically limits cellular biosynthesis given that it is a component of essential cellular building blocks including amino acids and nucleotides. Down-regulation of photosynthetic pathways may therefore serve to reduce cellular N demand. In conjunction with photosystem components (though not chl *a* metabolism), genes involved in mitochondrial respiration and in quenching of reactive oxygen species (ROS) were down-regulated. This is consistent with an overall picture of modulated metabolic activity in *S. trochoidea* in response to N-depletion also observed in other algae. For example, N and P limitation in the toxic alga *Prymnesium parvum* were found to decrease cytochrome and light harvesting gene expression in stationary growth (Beszteri et al., 2012), while N-starvation leads to four-fold down-regulation of genes related to the light harvesting complex in *Emiliana huxleyi* (Dyhrman et al., 2006). A reduction in chlorophyll, related to a decrease in the number of PSII reaction centers, has also been observed in a range of other eukaryotic marine algae, including such as chlorophytes, diatoms, prymnesiophytes (Berges et al., 1996; Simionato et al., 2013), as well as *Synechococcus* (Görl et al., 1998; Simionato et al., 2013). Down regulation of transcripts in *S. trochoidea* encoding high-nitrogen containing proteins like the photosystem proteins may further reflect changes in cellular stoichiometry to conserve N resources. Proteomic studies of N-limitation in dinoflagellates have shown a similar to our observed transcriptome data with down-regulated processes like carbon fixation, photosynthesis (Lee et al., 2009; Zhang et al., 2015). Alternatively, down-regulations is perhaps a way to compensate for increases in cellular C:N ratios. Measurements in *S. trochoidea* and *A. fundyense*, for example, have suggested increased ratios of particulate organic carbon (POC) to organic nitrogen (PON) under N limitation (Eberlein et al., 2016).

### Secondary metabolism

Prior studies of *S. trochoidea* have not noted toxic compounds found in other dinoflagellates. In fact, the perceived lack of toxicity is routinely cited as the basis for using this organism as a non-toxic control when testing other dinoflagellates such as *Alexandrium* for toxin production (Hold et al., 2001; Smith et al., 2002). Contradictory to this assumption, two *S. trochoidea* isolates have been found to be lethal to Eastern Oysters and Northern Quahog larvae, though not to Sheepshead Minnows (Tang and Gobler, 2012) raising the potential of previously unrecognized toxicity. Tang and Gobler (2012) demonstrated that late exponential and stationary phase cultures induce greater mortality in juvenile shellfish than exponential growth cultures. Experimental treatments of bivalve larvae with live culture, dead (frozen, boiled), and cell-free supernatant suggested a physiochemical mechanisms of toxicity involving either secondary metabolites or chemical constitutes from within the algae. The requisite toxic compounds or secondary metabolites have not been identified to date.

Saxitoxin is well known to occur in both Cyanobacteria and dinoflagellates (Hackett et al., 2013) and the presence of Saxitoxin was correlated with the detection of the *sxtA* genes by PCR in stains of *A. tamarense* (Stüken et al., 2011), although *sxtA* genes were also detected in strains in which toxin was not found. Other non-toxic and toxic dinoflagellates also appear to have partial saxitoxin pathways, though it appears that homologs of the C-terminal of *sxtA* and *sxtG* genes are exclusively associated with toxic strains (Hackett et al., 2013). Contigs annotated as *sxtA* in *S. trochoidea* did not include a C-terminus with an aminotransferase domain, suggesting that *S. trochoidea* likely does not produce traditional Saxitoxin. The transcriptome also contains several homologs to NRPS, and hybrid polyketide/NRPS genes, suggesting previously unrecognized secondary metabolism. A bacterial source of requisite PKS/NRPS sequences cannot be completely excluded, given that PKS genes have previously been correlated with bacterial presence in dinoflagellate cultures (Snyder et al., 2005), but is unlikely here given selection for eukaryotic mRNA (see Discussion above). Their potential involvement in toxicity remains unclear, given that it is quite difficult to ascertain toxicity from gene sequences alone. A detailed metabolite analysis of *S. trochoidea* may therefore be warranted to identify potential toxic secondary metabolites as well as previously unrecognized toxins similar to Saxitoxin.

### Conclusion

Data collected through the MMETSP project (Keeling et al., 2014) are a valuable resource for the interpretation of molecular datasets collected from the environment. Here we describe the most complete transcriptome of the high-density bloom forming *S. trochoidea* to date and demonstrate that transcript abundances are significantly affected by N availability. Overall, observations point to *S. trochoidea*'s ability to flexibly adapt to variations in N availability. These adaptations likely play a central role in near coastal environments where DIN sources are rapidly depleted in bloom conditions. The ability of *S. trochoidea* to switch to a resource utilization pattern that includes DON compounds may help sustain blooms and serve as a model for other persistent red tide events. Transcriptome data further suggests that *S. trochoidea* needs to be reevaluated for the potential to produce toxic secondary metabolites, an observation that may significantly influence the way *S. trochoidea* blooms are understood.

## Author contributions

BW and GS secured funding. JC, BW, and GS designed experiments. JC, BW, and GS interpreted the data. JC, BW, GS wrote the manuscript.

### Conflict of interest statement

The authors declare that the research was conducted in the absence of any commercial or financial relationships that could be construed as a potential conflict of interest.
