# Supplementary material for: Transcriptome Analysis of Scrippsiella trochoidea CCMP 3099 Reveals Physiological Changes Related to Nitrate Depletion
Source: Front Microbiol. 2016 May 9;7:639. doi: 10.3389/fmicb.2016.00639 (PMC4860509; doi:10.3389/fmicb.2016.00639)
Supplement: Supplementary file 1 [file DataSheet1.docx]

**SUPPLEMENTAL INFORMATION**

**Supplemental Table 1.** Nutrient concentrations at time of sampling.

|  | **Replete** | **Nitrogen-Limited** | **Phosphorus-Limited** |
| --- | --- | --- | --- |
| NO_x_ [μM] | 1759.6 (-4.5) | BD | 1020.3 (-8.4) |
| PO_4_ [μM] | 11.3 (2.0) | 18.7 (0.5) | 4.4 (1.9) |

Mean + sd, BD (Below detection)

**Supplemental Table 2.** Overall Result of the Illumina Transcriptome Sequencing

| Raw Data |  | Library | Data (Bp) | GC% | Mb |
| --- | --- | --- | --- | --- | --- |
|  | MMETSP0270 | Replete | 36,147,274 | 58 | 36.15 |
|  | MMETSP0271 | Nitrogen-limited | 36,131,119 | 58 | 36.13 |
|  | MMETSP0272 | Phosphorus-limited | 30,815,547 | 58 | 30.82 |
|  |  |  |  |  |  |
| Trimmed |  | Library | Data (Bp) | GC% | Mb |
|  | MMETSP0270 | Replete | 35,788,801 | 58 | 35.79 |
|  | MMETSP0271 | Nitrogen-limited | 35,770,337 | 58 | 35.77 |
|  | MMETSP0272 | Phosphorus-limited | 30,507,209 | 58 | 30.51 |

**Supplemental Table 3.** Summary of the transcriptome reduction procedure using CAP3 and CD-HIT-EST of *Scrippsiella trochoidea*, to collapse potential assembly artifacts using the Multi-*k*-mer assembly approach.

|  | **Assembly Name** | | |
| --- | --- | --- | --- |
|  | M-k Assembly | M-k Assembly + CAP3 | **M-k Assembly + CAP3 + CDHIT-EST** |
| Total  Length (bp) | 201,998,239 | 132,039,060 | **125,022,445** |
| Total  Contigs > 200 | 205,934 | 118,132 | **107,473** |
| Median  Contig size | 686 | 821 | **884** |
| Mean  Contig size | 980 | 1,117 | **1,163** |
| Maximum  Contig size | 25,590 | 26,110 | **26,110** |
| N50 Contig | 43,657 | 25,143 | **23,394** |
| N50 Length | 1,440 | 1,678 | **1,714** |
| N90 Length | 443 | 516 | **555** |

**Supplemental Table 4.** Annotation Results of the *S. trochoidea* transcriptome.

| **Annotation Pipeline** | **# Annotated Contigs** | **% Annotated Contigs** |
| --- | --- | --- |
| NCBI-NR database | 44,055 | 41.0% |
| Uniprot-SwissProt+TREMBL | 43,796 | 40.8% |
|  |  |  |
| Gene-Ontology Hits | 34,480 | 40.4% |
| GO:Cellular Component | 23,159 | 21.6% |
| GO:Biological Component | 30,453 | 28.4% |
| GO:Molecular Component | 24,415 | 22.7% |
|  |  |  |
| KEGG Matches | 29,489 | 27.5% |
| KEGG:GO | 16,148 | 15.0% |
| KOG Matches | 23,462 | 21.8% |
| Pfam-A matches | 36,749 | 34.2% |
| TIGRFAM matches | 3,098 | 2.9% |
|  |  |  |
| **Total Descriptions after filtering** | **43,785** | 40.7% |
| **Total Transcriptome** | **107,417** |  |

**Supplemental Table 5**. Average number of CEGS in sequenced protists genomes is lower than that of protistian transcriptome studies along with lower average number of duplicated orthologs (Average # of orthologs per CEG). Several genomes reach close to 95% partial completeness of the 248 ultraconserved CEGS, while transcriptomes reach 90%. Results of the CEGMA analysis suggest that current high throughout-put transcriptome studies recover almost complete reconstructions of key eukaryotic genes. Transcriptomes of protists also show either multiple transcripts coding for orthologs are present, as opposed to genomes where single orthologs are represented by single genes.

| Taxon | Sequencing  Type | Number of  248 CEGS | | Percent CEG  Completeness | | Average # of  orthologs per CEG | | % CEGS with  > 1 Ortholog | | #  Scaffolds | Study |
| --- | --- | --- | --- | --- | --- | --- | --- | --- | --- | --- | --- |
|  |  | Complete | Partial | Complete | Partial | Complete | Partial | Complete | Partial |  |  |
| *Phytophthora ramorum v1.1* | Genome | 229 | 236 | 92.34 | 95.16 | 1.18 | 1.26 | 15.28 | 20.34 | 2,576 | (Tyler et al., 2006) |
| *Phaeodactylum tricornutum* | Genome | 226 | 277 | 91.13 | 93.15 | 1.23 | 1.28 | 16.81 | 20.78 | 33 | (Bowler et al., 2008) |
| *Monosiga brevicollis* | Genome | 226 | 231 | 91.13 | 93.15 | 1.09 | 1.13 | 7.69 | 11.69 | 218 | (King et al., 2008) |
| *Thalassiosira pseudonana* | Genome | 225 | 230 | 90.73 | 92.74 | 1.24 | 1.32 | 18.67 | 23.04 | 27 | (Armbrust et al., 2004;Bowler et al., 2008) |
| *Dictyostelium purpureum QXDP1* | Genome | 223 | 233 | 89.92 | 93.95 | 1.20 | 1.24 | 15.70 | 19.31 | 799 | (Sucgang et al., 2011) |
| *Naegleria gruberi* | Genome | 220 | 225 | 88.71 | 90.73 | 1.24 | 1.32 | 15.91 | 20.89 | 784 | (Fritz-Laylin et al., 2010) |
| *Emiliania huxleyi* | Genome | 204 | 219 | 82.26 | 88.31 | 1.30 | 1.42 | 22.06 | 28.31 | 7,809 | (Read et al., 2013) |
| *Aureococcus anophagefferens* | Genome | 204 | 219 | 82.26 | 88.31 | 1.30 | 1.42 | 22.06 | 28.31 | 1,185 | (Gobler et al., 2011) |
| *Guillardia theta CCMP2712* | Genome | 202 | 209 | 81.45 | 84.27 | 1.22 | 1.30 | 14.85 | 20.10 | 670 | (Curtis et al., 2012) |
| *Bigelowiella natans CCMP2755* | Genome | 178 | 197 | 71.77 | 79.44 | 1.29 | 1.34 | 22.47 | 25.38 | 302 | (Curtis et al., 2012) |
| *Phytophthora capsici LT1534 v11.0* | Genome | 234 | 239 | 94.50 | 96.37 | 1.19 | 1.25 | 16.24 | 19.25 | 917 | (Lamour et al., 2012) |
| ***Scrippsiella trochoidea*** | **Transcriptome** | **210** | **220** | **85** | **89** | **2.74** | **2.93** | **81** | **82** | **107,417** | **This study** |

**Supplemental Table 6.** GO terms of genes detected as differentially expressed and down-regulated with a Log2 fold change lower than 0 of the N-limited vs replete.

| **GO term** | **Description** | **Annotated** | **Significant** | **Expected** | ***P-value*** |
| --- | --- | --- | --- | --- | --- |
| GO:0022900 | Electron transport chain | 162 | 8 | 0.47 | 5.70E-08 |
| GO:0015979 | Photosynthesis | 619 | 9 | 1.79 | 2.20E-05 |
| GO:0009767 | Photosynthetic electron transport chain | 52 | 6 | 0.15 | 6.30E-05 |
| GO:0018298 | Protein-chromophore linkage | 274 | 6 | 0.79 | 7.20E-05 |
| GO:0006351 | Transcription, DNA-templated | 1394 | 8 | 4.04 | 1.10E-04 |
| GO:0009636 | Response to toxic substance | 153 | 4 | 0.44 | 3.20E-04 |
| GO:0044271 | Cellular nitrogen compound biosynthetic process | 2530 | 12 | 7.33 | 3.20E-04 |
| GO:2000112 | Regulation of cellular macromolecule biosynthetic process | 1850 | 9 | 5.36 | 4.40E-04 |
| GO:0006091 | Generation of precursor metabolites and energy | 933 | 9 | 2.7 | 5.20E-04 |
| GO:0055114 | Oxidation-reduction process | 902 | 10 | 2.61 | 5.60E-04 |
| GO:0018130 | Heterocycle biosynthetic process | 2415 | 11 | 7 | 1.22E-03 |
| GO:0019438 | Aromatic compound biosynthetic process | 2444 | 11 | 7.08 | 1.27E-03 |
| GO:0009064 | Glutamine family amino acid metabolic process | 171 | 5 | 0.5 | 1.49E-03 |
| GO:1901362 | Organic cyclic compound biosynthetic process | 2620 | 12 | 7.59 | 1.83E-03 |
| GO:0032774 | RNA biosynthetic process | 1424 | 8 | 4.12 | 2.17E-03 |
| GO:0010556 | Regulation of macromolecule biosynthetic process | 1870 | 9 | 5.42 | 2.27E-03 |
| GO:0006811 | Ion transport | 2297 | 17 | 6.65 | 2.32E-03 |
| GO:0030509 | BMP signaling pathway | 20 | 2 | 0.06 | 2.47E-03 |
| GO:0051340 | Regulation of ligase activity | 31 | 2 | 0.09 | 3.54E-03 |
| GO:0015849 | Organic acid transport | 268 | 5 | 0.78 | 3.96E-03 |
| GO:0071705 | Nitrogen compound transport | 591 | 7 | 1.71 | 4.08E-03 |
| GO:0035335 | Peptidyl-tyrosine dephosphorylation | 17 | 2 | 0.05 | 4.90E-03 |
| GO:0060255 | Regulation of macromolecule metabolic process | 2824 | 11 | 8.18 | 5.79E-03 |
| GO:0000041 | Transition metal ion transport | 78 | 3 | 0.23 | 6.09E-03 |
| GO:1903320 | Regulation of protein modification by small protein conjugation or removal | 139 | 3 | 0.4 | 6.75E-03 |
| GO:0031396 | Regulation of protein ubiquitination | 125 | 3 | 0.36 | 7.52E-03 |
| GO:0051252 | Regulation of RNA metabolic process | 1393 | 8 | 4.03 | 7.88E-03 |
| GO:0045165 | Cell fate commitment | 136 | 2 | 0.39 | 9.58E-03 |
| GO:0034654 | Nucleobase-containing compound biosynthetic process | 2091 | 11 | 6.06 | 1.01E-02 |
| GO:0010468 | Regulation of gene expression | 2222 | 9 | 6.44 | 1.12E-02 |
| GO:0031326 | Regulation of cellular biosynthetic process | 2000 | 9 | 5.79 | 1.18E-02 |
| GO:0051351 | Positive regulation of ligase activity | 23 | 2 | 0.07 | 1.19E-02 |
| GO:0031398 | Positive regulation of protein ubiquitination | 45 | 2 | 0.13 | 1.19E-02 |
| GO:1903322 | Positive regulation of protein modification by small protein conjugation or removal | 47 | 2 | 0.14 | 1.21E-02 |
| GO:0006464 | Cellular protein modification process | 3500 | 11 | 10.14 | 1.24E-02 |
| GO:0006865 | Amino acid transport | 169 | 5 | 0.49 | 1.25E-02 |
| GO:0006820 | Anion transport | 578 | 9 | 1.67 | 1.26E-02 |
| GO:0071941 | Nitrogen cycle metabolic process | 58 | 2 | 0.17 | 1.36E-02 |
| GO:0036211 | Protein modification process | 3500 | 11 | 10.14 | 1.50E-02 |
| GO:0090092 | Regulation of transmembrane receptor protein serine/threonine kinase signaling pathway | 37 | 2 | 0.11 | 1.54E-02 |
| GO:0009889 | Regulation of biosynthetic process | 2016 | 9 | 5.84 | 1.65E-02 |
| GO:0044699 | Single-organism process | 15761 | 54 | 45.65 | 1.67E-02 |
| GO:0015711 | Organic anion transport | 392 | 5 | 1.14 | 1.79E-02 |
| GO:0048870 | Cell motility | 520 | 4 | 1.51 | 1.93E-02 |
| GO:0051438 | Regulation of ubiquitin-protein transferase activity | 31 | 2 | 0.09 | 1.95E-02 |
| GO:0060674 | Placenta blood vessel development | 13 | 1 | 0.04 | 2.07E-02 |
| GO:0072006 | Nephron development | 17 | 1 | 0.05 | 2.08E-02 |
| GO:0061053 | Somite development | 19 | 1 | 0.06 | 2.17E-02 |
| GO:0045637 | Regulation of myeloid cell differentiation | 27 | 1 | 0.08 | 2.80E-02 |
| GO:0006355 | Regulation of transcription, DNA-templated | 1276 | 8 | 3.7 | 2.85E-02 |
| GO:0060411 | Cardiac septum morphogenesis | 24 | 1 | 0.07 | 2.92E-02 |
| GO:0051443 | Positive regulation of ubiquitin-protein transferase activity | 23 | 2 | 0.07 | 3.03E-02 |
| GO:1902105 | Regulation of leukocyte differentiation | 29 | 1 | 0.08 | 3.19E-02 |
| GO:0032786 | Positive regulation of DNA-templated transcription, elongation | 15 | 1 | 0.04 | 3.29E-02 |
| GO:0003206 | Cardiac chamber morphogenesis | 29 | 1 | 0.08 | 3.52E-02 |
| GO:0007178 | Transmembrane receptor protein serine/threonine kinase signaling pathway | 49 | 2 | 0.14 | 3.60E-02 |
| GO:0010632 | Regulation of epithelial cell migration | 15 | 1 | 0.04 | 3.63E-02 |
| GO:0034329 | Cell junction assembly | 61 | 1 | 0.18 | 3.78E-02 |
| GO:0051347 | Positive regulation of transferase activity | 194 | 3 | 0.56 | 3.82E-02 |
| GO:0080090 | Regulation of primary metabolic process | 2887 | 11 | 8.36 | 3.91E-02 |
| GO:0001756 | Somitogenesis | 18 | 1 | 0.05 | 3.92E-02 |
| GO:0043085 | Positive regulation of catalytic activity | 343 | 3 | 0.99 | 4.00E-02 |
| GO:0019219 | Regulation of nucleobase-containing compound metabolic process | 1766 | 8 | 5.12 | 4.16E-02 |
| GO:0000077 | DNA damage checkpoint | 93 | 2 | 0.27 | 4.17E-02 |
| GO:0032270 | Positive regulation of cellular protein metabolic process | 430 | 3 | 1.25 | 4.30E-02 |
| GO:0006520 | Cellular amino acid metabolic process | 901 | 5 | 2.61 | 4.33E-02 |
| GO:0019684 | Photosynthesis, light reaction | 322 | 6 | 0.93 | 4.58E-02 |
| GO:0046034 | ATP metabolic process | 528 | 4 | 1.53 | 4.67E-02 |
| GO:0051179 | Localization | 5692 | 23 | 16.49 | 4.74E-02 |

**Supplemental Table 7.** GO terms of genes detected as differentially expressed and up-regulated with a Log2 fold change lower than 0 of the N-limited vs Replete.

| **GO term** | **Description** | **Annotated** | **Significant** | **Expected** | **P-value** |
| --- | --- | --- | --- | --- | --- |
| GO:0071705 | Nitrogen compound transport | 591 | 8 | 1.35 | 3.10E-04 |
| GO:0044711 | Single-organism biosynthetic process | 2748 | 11 | 6.3 | 3.90E-04 |
| GO:0019682 | Glyceraldehyde-3-phosphate metabolic process | 17 | 2 | 0.04 | 1.22E-03 |
| GO:0008299 | Isoprenoid biosynthetic process | 120 | 3 | 0.27 | 1.41E-03 |
| GO:0019288 | Isopentenyl diphosphate biosynthetic process, methylerythritol 4-phosphate pathway | 14 | 2 | 0.03 | 1.91E-03 |
| GO:0046490 | Isopentenyl diphosphate metabolic process | 18 | 2 | 0.04 | 2.28E-03 |
| GO:0044264 | Cellular polysaccharide metabolic process | 549 | 4 | 1.26 | 2.71E-03 |
| GO:0007588 | Excretion | 35 | 2 | 0.08 | 3.49E-03 |
| GO:0090407 | Organophosphate biosynthetic process | 831 | 6 | 1.9 | 3.67E-03 |
| GO:0042886 | Amide transport | 107 | 3 | 0.25 | 7.21E-03 |
| GO:0006793 | Phosphorus metabolic process | 3410 | 12 | 7.81 | 7.71E-03 |
| GO:0051189 | Prosthetic group metabolic process | 14 | 1 | 0.03 | 1.05E-02 |
| GO:0015833 | Peptide transport | 93 | 3 | 0.21 | 1.07E-02 |
| GO:0009240 | Isopentenyl diphosphate biosynthetic process | 18 | 2 | 0.04 | 1.23E-02 |
| GO:0006777 | Mo-molybdopterin cofactor biosynthetic process | 14 | 1 | 0.03 | 1.46E-02 |
| GO:0040008 | Regulation of growth | 387 | 3 | 0.89 | 1.48E-02 |
| GO:0006857 | Oligopeptide transport | 24 | 3 | 0.05 | 1.56E-02 |
| GO:0005976 | Polysaccharide metabolic process | 695 | 4 | 1.59 | 1.60E-02 |
| GO:0006081 | Cellular aldehyde metabolic process | 96 | 2 | 0.22 | 1.62E-02 |
| GO:0006720 | Isoprenoid metabolic process | 168 | 3 | 0.38 | 1.68E-02 |
| GO:0001558 | Regulation of cell growth | 141 | 2 | 0.32 | 1.86E-02 |
| GO:0044765 | Single-organism transport | 4055 | 16 | 9.29 | 1.94E-02 |
| GO:0008610 | Lipid biosynthetic process | 902 | 5 | 2.07 | 1.99E-02 |
| GO:0000272 | Polysaccharide catabolic process | 402 | 3 | 0.92 | 2.02E-02 |
| GO:0009726 | Detection of endogenous stimulus | 24 | 1 | 0.05 | 2.14E-02 |
| GO:0009403 | Toxin biosynthetic process | 11 | 1 | 0.03 | 2.15E-02 |
| GO:0000281 | Mitotic cytokinesis | 151 | 2 | 0.35 | 2.25E-02 |
| GO:0044281 | Small molecule metabolic process | 4226 | 12 | 9.68 | 2.36E-02 |
| GO:0021984 | Adenohypophysis development | 15 | 1 | 0.03 | 2.56E-02 |
| GO:0043545 | Molybdopterin cofactor metabolic process | 14 | 1 | 0.03 | 2.60E-02 |
| GO:1901463 | Regulation of tetrapyrrole biosynthetic process | 15 | 1 | 0.03 | 2.77E-02 |
| GO:1901401 | Regulation of tetrapyrrole metabolic process | 15 | 1 | 0.03 | 2.81E-02 |
| GO:0030435 | Sporulation resulting in formation of a cellular spore | 170 | 2 | 0.39 | 2.81E-02 |
| GO:0035270 | Endocrine system development | 19 | 1 | 0.04 | 2.86E-02 |
| GO:0043934 | Sporulation | 171 | 2 | 0.39 | 3.25E-02 |
| GO:0019637 | Organophosphate metabolic process | 1984 | 8 | 4.55 | 3.33E-02 |
| GO:0045329 | Carnitine biosynthetic process | 11 | 1 | 0.03 | 3.43E-02 |
| GO:1901135 | Carbohydrate derivative metabolic process | 2059 | 8 | 4.72 | 3.51E-02 |
| GO:0051785 | Positive regulation of nuclear division | 200 | 2 | 0.46 | 3.57E-02 |
| GO:0021536 | Diencephalon development | 22 | 1 | 0.05 | 3.74E-02 |
| GO:0032324 | Molybdopterin cofactor biosynthetic process | 14 | 1 | 0.03 | 3.85E-02 |
| GO:0010288 | Response to lead ion | 16 | 1 | 0.04 | 3.87E-02 |
| GO:0070589 | Cellular component macromolecule biosynthetic process | 57 | 1 | 0.13 | 3.89E-02 |
| GO:0045981 | Positive regulation of nucleotide metabolic process | 15 | 1 | 0.03 | 4.25E-02 |
| GO:0008654 | Phospholipid biosynthetic process | 176 | 2 | 0.4 | 4.29E-02 |
| GO:0051193 | Regulation of cofactor metabolic process | 26 | 1 | 0.06 | 4.33E-02 |
| GO:0030810 | Positive regulation of nucleotide biosynthetic process | 15 | 1 | 0.03 | 4.34E-02 |
| GO:0065002 | Intracellular protein transmembrane transport | 50 | 1 | 0.11 | 4.36E-02 |
| GO:0016525 | Negative regulation of angiogenesis | 12 | 1 | 0.03 | 4.48E-02 |
| GO:0071702 | Organic substance transport | 2099 | 11 | 4.81 | 4.56E-02 |
| GO:0030308 | Negative regulation of cell growth | 32 | 1 | 0.07 | 4.71E-02 |
| GO:0007589 | Body fluid secretion | 25 | 1 | 0.06 | 4.80E-02 |
| GO:0051783 | Regulation of nuclear division | 285 | 2 | 0.65 | 4.82E-02 |
| GO:0042440 | Pigment metabolic process | 190 | 2 | 0.44 | 4.84E-02 |

**Supplemental Table 8:** Differentially expressed genes detected in the N-limited vs replete. Grouped by category based on KEGG and GO terms from the annotation. Genes sorted in each category by the Log2 Fold change as a measure of relative gene expression, >0 up-regulated < 0 down-regulated.

| **Photosynthetic Pathways and Electron Chain Transport** | | | | |  |  |
| --- | --- | --- | --- | --- | --- | --- |
| Contig Name | Contig  Length | Log2  Fold Change | *P*-adjusted | Description | Best Blastx NR Hit Organism ID | Blast Hit  E-value |
| ScrippsTroch_8612 | 1704 | -2.63 | 2.62E-69 | Photosystem II CP47 chlorophyll apoprotein | *Heterocapsa triquetra* | 0.00E+00 |
| ScrippsTroch_10865 | 2323 | -2.31 | 1.81E-53 | ATP synthase subunit beta, chloroplastic | *Prorocentrum minimum* | 5.26E-180 |
| ScrippsTroch_15482 | 2316 | -2.05 | 1.10E-42 | Photosystem I P700 chlorophyll a apoprotein A2 | *Heterocapsa triquetra* | 0.00E+00 |
| ScrippsTroch_9027 | 2263 | -2.05 | 1.04E-42 | Photosystem I P700 chlorophyll a apoprotein A1 | *Heterocapsa triquetra* | 0.00E+00 |
| ScrippsTroch_93030 | 1219 | -1.95 | 1.18E-06 | Opioid growth factor receptor (OGFr) conserved region | *Nostoc punctiforme* PCC 73102 | 9.08E-26 |
| ScrippsTroch_680 | 1637 | -1.88 | 1.80E-36 | Photosystem II CP43 chlorophyll apoprotein | *Heterocapsa triquetra* | 0.00E+00 |
| ScrippsTroch_14964 | 1247 | -1.81 | 3.36E-35 | Photosystem II D2 protein | *Lingulodinium polyedrum* | 0.00E+00 |
| ScrippsTroch_103721 | 1507 | -1.77 | 2.95E-27 | ATP synthase subunit alpha, chloroplastic | *Heterocapsa triquetra* | 0.00E+00 |
| ScrippsTroch_36534 | 1085 | -1.75 | 1.14E-04 | Phototropin-2 | *Aureococcus anophagefferens* | 1.20E-12 |
| ScrippsTroch_107426 | 204 | -1.51 | 6.35E-19 | Photosystem Q(B) protein | *Symbiodinium sp.* D1a | 4.84E-35 |
| ScrippsTroch_1641 | 1169 | -1.49 | 7.40E-14 | Flavodoxin | *Thalassiosira oceanica* | 1.34E-57 |
| ScrippsTroch_7736 | 517 | -1.37 | 3.10E-16 | Cytochrome b6-f complex subunit 4 | *Heterocapsa triquetra* | 1.82E-66 |
| ScrippsTroch_23237 | 893 | -1.02 | 6.21E-09 | Cytochrome b6 | *Vaucheria litorea* | 8.03E-116 |
| ScrippsTroch_67264 | 338 | -0.71 | 4.27E-04 | Cytochrome b559 subunit alpha | *Heterocapsa triquetra* | 2.77E-24 |
|  |  |  |  |  |  |  |
| **Aerobic Respiratory Pathways (Mitochondrion) and Oxidative Phosphorylation** | | | | |  |  |
| Contig Name | Contig  Length | Log2  Fold Change | *P*-adjusted | Description | Best Blastx NR Hit Organism ID | Blast Hit  E-value |
| ScrippsTroch_44656 | 1328 | -2.96 | 4.28E-19 | Cytochrome c peroxidase, mitochondrial | *Symbiodinium sp.* clade C | 1.01E-79 |
| ScrippsTroch_2154 | 1526 | -0.84 | 1.07E-04 | Cytochrome b | *Alexandrium catenella* | 0.00E+00 |
| ScrippsTroch_41606 | 2927 | -0.84 | 5.94E-03 | Uncharacterized protein | *Oxytricha trifallax* | 3.43E-06 |
| ScrippsTroch_10428 | 2005 | -0.78 | 1.78E-05 | Cytochrome c oxidase subunit 1 | *Pfiesteria piscicida* | 0.00E+00 |
| ScrippsTroch_12365 | 1044 | -0.74 | 7.26E-02 | Glutathione S-transferase F10 | *Guillardia theta* CCMP2712 | 2.57E-20 |
| ScrippsTroch_82782 | 3961 | -0.74 | 4.98E-02 | ATP-binding cassette transporter abc2 | *Aureococcus anophagefferens* | 1.65E-114 |
| ScrippsTroch_766 | 2801 | 0.60 | 9.15E-02 | Pyrophosphate-energized vacuolar membrane proton pump | *Perkinsus marinus* ATCC 50983 | 0.00E+00 |
| ScrippsTroch_756 | 2598 | 0.66 | 6.65E-03 | Plasma membrane ATPase 2 | *Emiliania huxleyi* CCMP1516 | 0.00E+00 |
| ScrippsTroch_97018 | 2596 | 0.99 | 2.42E-02 | ABC transporter B family member 25, mitochondrial | *Azospirillum brasilense* | 6.57E-90 |
| ScrippsTroch_94131 | 1901 | 1.06 | 2.87E-02 | Blue copper oxidase CueO | *Ectocarpus siliculosus* | 2.93E-43 |
| ScrippsTroch_95319 | 1904 | 1.58 | 2.88E-03 | Cytochrome P450 CYP12A2 | *Emiliania huxleyi* CCMP1516 | 1.81E-61 |
| ScrippsTroch_35953 | 1869 | 1.87 | 7.21E-07 | Carotenoid 9,10(9',10')-cleavage dioxygenase 1 | *Cyanothece sp.* ATCC 51142 | 4.77E-92 |
|  |  |  |  |  |  |  |
| **Amino Acid Biosynthesis, Catabolism, Transport and Nucleotide Metabolism** | | | | |  |  |
| Contig Name | Contig  Length | Log2  Fold Change | *P*-adjusted | Description | Best Blastx NR Hit Organism ID | Blast Hit  E-value |
| ScrippsTroch_22942 | 1511 | -3.67 | 3.77E-39 | Putative glutamine amidotransferase-like protein RP404 | *Setaria italica* | 9.71E-79 |
| ScrippsTroch_11517 | 2026 | -2.56 | 1.50E-31 | Urea transporter 2 | *Thalassiosira pseudonana* CCMP1335 | 2.37E-28 |
| ScrippsTroch_54819 | 2208 | -1.83 | 8.25E-17 | Ammonium transporter 1 member 2 | *Emiliania huxleyi* CCMP1516 | 8.14E-81 |
| ScrippsTroch_31305 | 1330 | -1.33 | 3.78E-03 | Arginase | *Perkinsus marinus* ATCC 50983 | 7.47E-102 |
| ScrippsTroch_75181 | 1520 | -1.31 | 3.13E-07 | Putative glutamine amidotransferase-like protein RP404 | *Zea mays* | 7.44E-77 |
| ScrippsTroch_7564 | 1757 | -1.16 | 4.74E-02 | Ammonia channel | *Micromonas sp.* RCC299 | 1.76E-111 |
| ScrippsTroch_32806 | 3396 | -1.11 | 1.39E-04 | Glutamate dehydrogenase 2 | *Perkinsus marinus* ATCC 50983 | 0.00E+00 |
| ScrippsTroch_21007 | 1884 | -1.09 | 5.48E-06 | Ammonia channel | *Desulfosporosinus sp.* OT | 2.85E-104 |
| ScrippsTroch_64338 | 4584 | -0.96 | 2.60E-06 | Leu/Ile/Val-binding protein homolog 5 | *Emiliania huxleyi* CCMP1516 | 8.59E-45 |
| ScrippsTroch_76491 | 2248 | -0.95 | 7.26E-02 | Putative serine/threonine-protein kinase/receptor R831 | *Dictyostelium purpureum* | 1.69E-30 |
| ScrippsTroch_853 | 1026 | -0.93 | 5.83E-06 | Leu/Ile/Val-binding protein homolog 5 | *Thermodesulfovibrio yellowstonii* DSM 11347 | 3.17E-32 |
| ScrippsTroch_92135 | 1931 | -0.89 | 4.59E-02 | Vacuolar amino acid transporter, putative | *Perkinsus marinus* ATCC 50983 | 1.67E-09 |
| ScrippsTroch_18019 | 3966 | -0.80 | 1.37E-05 | Leu/Ile/Val-binding protein homolog 5 | *Desulfurobacterium sp.* TC5-1 | 1.52E-42 |
| ScrippsTroch_15563 | 628 | -0.80 | 5.78E-03 | Putative ammonium transporter 1 | *Perkinsus marinus* ATCC 50983 | 6.89E-23 |
| ScrippsTroch_24733 | 2294 | -0.76 | 1.14E-04 | Nitrate/nitrite transporter NarK | *Alkalilimnicola ehrlichii* MLHE-1 | 2.45E-105 |
| ScrippsTroch_22355 | 5900 | -0.68 | 2.38E-03 | Glutamate synthase [NADH], amyloplastic | *Theobroma cacao* | 0.00E+00 |
| ScrippsTroch_3948 | 4388 | -0.66 | 1.72E-02 | Leu/Ile/Val-binding protein homolog 5 | *Desulfurobacterium sp.* TC5-1 | 5.64E-42 |
| ScrippsTroch_15151 | 2211 | 0.61 | 1.81E-02 | Extracellular serine proteinase | *Thermus sp.* CCB_US3_UF1 | 2.12E-61 |
| ScrippsTroch_21629 | 991 | 0.81 | 4.17E-03 | Alkaline phosphatase | *Amphidinium carterae* | 5.44E-75 |
| ScrippsTroch_2948 | 1718 | 0.86 | 2.41E-02 | Alkaline phosphatase | *Amphidinium carterae* | 3.83E-99 |
| ScrippsTroch_27837 | 2360 | 0.88 | 5.18E-02 | Uric acid-xanthine permease | *Aureococcus anophagefferens* | 0.00E+00 |
| ScrippsTroch_99737 | 3566 | 1.04 | 2.51E-03 | Atrial natriuretic peptide receptor 1 | *Saprolegnia diclina* VS20 | 2.22E-30 |
| ScrippsTroch_106819 | 297 | 1.06 | 2.39E-02 | Glr3054 protein | *Mastigocladopsis repens* | 5.29E-15 |
| ScrippsTroch_11718 | 2313 | 1.07 | 9.26E-06 | Oligopeptide transporter 6 | *Rhizopus delemar* RA 99-880 | 1.46E-73 |
| ScrippsTroch_38152 | 2066 | 1.13 | 3.31E-02 | Xanthine/uracil/vitamin C permease | *Micromonas sp*. RCC299 | 8.63E-165 |
| ScrippsTroch_68626 | 1792 | 1.17 | 9.52E-02 | Probable aspartyl aminopeptidase | *Toxoplasma gondii* GT1 | 3.42E-147 |
| ScrippsTroch_49232 | 2661 | 1.26 | 5.97E-04 | Protein-glutamine gamma-glutamyltransferase 4 | *Strongylocentrotus purpuratus* | 7.07E-45 |
| ScrippsTroch_81896 | 2392 | 1.31 | 3.62E-02 | Polyamine oxidase | *Branchiostoma floridae* | 6.46E-67 |
| ScrippsTroch_58245 | 1849 | 1.36 | 5.15E-02 | Protein NRT1/ PTR FAMILY 8.1 | *Emiliania huxleyi* CCMP1516 | 4.62E-137 |
| ScrippsTroch_31391 | 1508 | 1.46 | 9.09E-02 | CTP synthase | *Haloferax mucosum* | 1.02E-107 |
| ScrippsTroch_98466 | 1856 | 1.62 | 6.38E-06 | Ammonium transporter 2 | *Perkinsus marinus* ATCC 50983 | 1.24E-119 |
| ScrippsTroch_47326 | 1281 | 1.87 | 5.94E-03 | Protein NRT1/ PTR FAMILY 8.1 | *Emiliania huxleyi* CCMP1516 | 9.12E-42 |
| ScrippsTroch_53972 | 848 | 2.09 | 3.54E-02 | Probable serine/threonine-protein kinase DDB_G0286627 | *Paramecium tetraurelia* strain d4-2 | 1.99E-12 |
| ScrippsTroch_13522 | 4695 | 2.18 | 5.72E-18 | Aliphatic amidase expression-regulating protein | *Moorea producens* | 1.22E-156 |
| ScrippsTroch_73524 | 1668 | 2.42 | 4.88E-07 | Putative ammonium transporter MTH_663 | *Parvibaculum lavamentivorans* DS-1 | 3.80E-73 |
| ScrippsTroch_38956 | 1747 | 4.05 | 1.15E-22 | hypothetical protein | *Paramecium tetraurelia* strain d4-2 | 8.35E-09 |
| ScrippsTroch_70431 | 1806 | 4.12 | 3.87E-18 | Probable gamma-butyrobetaine dioxygenase | *Aureococcus anophagefferens* | 1.43E-106 |
| ScrippsTroch_49680 | 1771 | 5.39 | 7.12E-32 | Proton-coupled amino acid transporter 4 | *Emiliania huxleyi* CCMP1516 | 2.47E-21 |
|  |  |  |  |  |  |  |
| **Carbohydrate and Lipid Metabolism** | | |  |  |  |  |
| Contig Name | Contig  Length | Log2  Fold Change | *P*-adjusted | Description | Best Blastx NR Hit Organism ID | Blast Hit  E-value |
| ScrippsTroch_72402 | 1901 | -2.47 | 2.59E-13 | L-serine dehydratase | *Acinetobacter sp.* NIPH 542 | 3.99E-116 |
| ScrippsTroch_15569 | 890 | -0.73 | 9.40E-03 | Sterol 3-beta-glucosyltransferase UGT80A2 | *Arabidopsis thaliana* | 2.76E-23 |
| ScrippsTroch_10678 | 5877 | -0.67 | 8.32E-02 | PBS lyase HEAT-like repeat protein | *Geitlerinema sp.* PCC 7105 | 1.68E-21 |
| ScrippsTroch_20152 | 1740 | 0.72 | 1.95E-02 | Glucan 1,3-beta-glucosidase | *Ostreococcus tauri* | 1.56E-59 |
| ScrippsTroch_10719 | 2223 | 0.79 | 3.68E-02 | Uncharacterized protein | *Emiliania huxleyi* CCMP1516 | 3.53E-35 |
| ScrippsTroch_1383 | 1274 | 0.97 | 6.78E-02 | Beta-glucanase | *Cellvibrio japonicus* Ueda107 | 4.06E-34 |
| ScrippsTroch_46275 | 2658 | 1.00 | 2.73E-02 | Processive diacylglycerol beta-glucosyltransferase | *Waddlia chondrophila* 2032/99 | 8.32E-21 |
| ScrippsTroch_96824 | 2928 | 1.01 | 4.96E-03 | Non-specific lipid-transfer protein | *Nematostella vectensis* | 3.82E-121 |
| ScrippsTroch_8125 | 2474 | 1.08 | 8.98E-05 | Endoglucanase-5 | *Aureococcus anophagefferens* | 1.23E-89 |
| ScrippsTroch_6050 | 1808 | 1.35 | 7.07E-05 | Uncharacterized protein R632 | *Salpingoeca rosetta* | 4.59E-26 |
| ScrippsTroch_76422 | 1657 | 1.42 | 2.20E-03 | Probable 1,4-beta-D-glucan cellobiohydrolase B | *Lingulodinium polyedrum* | 0.00E+00 |
| ScrippsTroch_27986 | 1770 | 1.42 | 3.81E-02 | Uncharacterized protein | *Emiliania huxleyi* CCMP1516 | 1.70E-61 |
| ScrippsTroch_6910 | 1122 | 1.55 | 6.99E-02 | Stearoyl-CoA 9-desaturase / linoleoyl-CoA desaturase | *Guillardia theta* CCMP2712 | 2.68E-86 |
| ScrippsTroch_1197 | 1846 | 1.60 | 5.76E-04 | UDP-galactose translocator | *Emiliania huxleyi* CCMP1516 | 1.82E-29 |
| ScrippsTroch_3215 | 1911 | 1.66 | 9.16E-13 | 1,4-beta-D-glucan cellobiohydrolase B | *Pyrocystis lunula* | 0.00E+00 |
|  |  |  |  |  |  |  |
| **Secondary Metabolites and Production of Terpenoids** | | | | |  |  |
| Contig Name | Contig  Length | Log2  Fold Change | *P*-adjusted | Description | Best Blastx NR Hit Organism ID | Blast Hit  E-value |
| ScrippsTroch_16047 | 5167 | 0.71 | 6.47E-03 | Chondramide synthase cmdD | *Scytonema hofmanni* | 9.87E-101 |
| ScrippsTroch_6515 | 4233 | 0.85 | 1.18E-02 | SxtA short isoform | *Alexandrium fundyense* | 1.04E-84 |
| ScrippsTroch_80487 | 1877 | 1.07 | 7.03E-02 | Probable zeta-carotene desaturase | *Saccoglossus kowalevskii* | 1.42E-155 |
| ScrippsTroch_32485 | 1596 | 1.50 | 3.18E-03 | Clavaminate synthase-like protein At3g21360 | *Saccoglossus kowalevskii* | 2.14E-42 |
| ScrippsTroch_503 | 1862 | 1.79 | 6.94E-08 | 1-deoxy-D-xylulose 5-phosphate reductoisomerase | *Pyrocystis lunula* | 0.00E+00 |
| ScrippsTroch_98501 | 872 | 2.51 | 1.08E-02 | 1-deoxy-D-xylulose 5-phosphate reductoisomerase | *Pyrocystis lunula* | 0.00E+00 |
| ScrippsTroch_6916 | 2449 | 2.61 | 1.43E-06 | Limonene hydroxylase | *Moorea producens* | 1.19E-67 |
|  |  |  |  |  |  |  |
| **Cell Cycle, Transcription, DNA Repair** | | |  |  |  |  |
| Contig Name | Contig  Length | Log2  Fold Change | *P*-adjusted | Description | Best Blastx NR Hit Organism ID | Blast Hit  E-value |
| ScrippsTroch_92158 | 2488 | -2.66 | 3.98E-12 | Cell division control protein 2 homolog | *Phytophthora parasitica* | 2.08E-36 |
| ScrippsTroch_93642 | 2045 | -2.28 | 1.31E-04 | Retrovirus-related Pol polyprotein from  type-1 retrotransposable element R2 | *Ancylostoma ceylanicum* | 2.48E-11 |
| ScrippsTroch_100526 | 1669 | -1.81 | 1.92E-10 | Dual specificity protein phosphatase CDC14B | *Vicugna pacos* | 1.66E-13 |
| ScrippsTroch_46515 | 788 | -1.50 | 1.15E-02 | Pumilio homolog 3 | *Piriformospora indica* DSM 11827 | 1.25E-19 |
| ScrippsTroch_43559 | 2287 | -1.07 | 2.54E-02 | Zinc finger CCCH domain-containing protein 39 | *Cryptosporidium parvum* Iowa II | 1.63E-19 |
| ScrippsTroch_1919 | 4343 | -1.04 | 8.20E-03 | GTPase IMAP family member 4-like | *Danio rerio* | 1.05E-16 |
| ScrippsTroch_23702 | 878 | -0.98 | 2.67E-05 | Signal peptide, CUB and EGF-like domain-containing protein 2 | *Cyanidioschyzon merolae* strain 10D | 1.13E-17 |
| ScrippsTroch_33935 | 1851 | -0.90 | 3.69E-02 | Dual specificity protein phosphatase CDC14B | *Falco peregrinus* | 2.70E-14 |
| ScrippsTroch_8531 | 1814 | -0.76 | 7.24E-02 | Zinc finger CCCH domain-containing protein 39 | *Cryptosporidium parvum* Iowa II | 3.65E-22 |
| ScrippsTroch_22187 | 2041 | -0.65 | 9.44E-02 | Mitogen-activated protein kinase homolog MMK1 | *Paramecium tetraurelia* strain d4-2 | 2.07E-84 |
| ScrippsTroch_8481 | 8288 | 0.60 | 5.45E-02 | Anguibactin system regulator | *Streptomyces collinus* Tu 365 | 6.36E-119 |
| ScrippsTroch_82689 | 1821 | 0.82 | 3.40E-02 | Uncharacterized protein C29B5.04c | *Schlesneria paludicola* | 1.51E-34 |
| ScrippsTroch_23316 | 3678 | 0.94 | 2.34E-06 | Subtilisin DY | *Moorea producens* | 1.95E-84 |
| ScrippsTroch_11636 | 1721 | 1.00 | 7.34E-03 | Cysteine-rich motor neuron 1 protein | *Caligus clemensi* | 2.13E-06 |
| ScrippsTroch_102995 | 1801 | 1.02 | 3.08E-02 | Spore coat protein A | *Bathycoccus prasinos* | 3.14E-54 |
| ScrippsTroch_49724 | 1562 | 1.07 | 7.48E-02 | Protein MEI2-like 4 | *Plasmodium chabaudi chabaudi* | 3.68E-29 |
| ScrippsTroch_13740 | 2694 | 1.20 | 1.52E-03 | Polycystin-2 | Guillardia theta CCMP2712 | 8.02E-11 |
| ScrippsTroch_58224 | 3279 | 1.21 | 2.53E-05 | Uncharacterized protein | *Emiliania huxleyi* CCMP1516 | 5.07E-42 |
| ScrippsTroch_20901 | 1558 | 1.22 | 9.49E-03 | Mitogen-activated protein kinase NPK1 | *Paramecium tetraurelia* strain d4-2 | 7.02E-43 |
| ScrippsTroch_91197 | 1534 | 1.28 | 2.93E-02 | Meiosis protein mei2 | *Plasmodium chabaudi chabaudi* | 1.05E-17 |
| ScrippsTroch_53034 | 1708 | 1.30 | 6.06E-02 | Endonuclease/exonuclease/phosphatase | *Streptomyces sviceus* | 2.45E-24 |
| ScrippsTroch_44948 | 1967 | 1.62 | 1.83E-05 | Uncharacterized protein | *Polysphondylium pallidum* PN500 | 3.61E-91 |
|  |  |  |  |  |  |  |
| **Endoplasmic Reticulum, Proteosome, Membranes** | | | |  |  |  |
| Contig Name | Contig  Length | Log2  Fold Change | *P*-adjusted | Description | Best Blastx NR Hit Organism ID | Blast Hit  E-value |
| ScrippsTroch_87206 | 2324 | -1.27 | 3.48E-03 | Lysosomal Pro-X carboxypeptidase | *Emiliania huxleyi* CCMP1516 | 1.22E-72 |
| ScrippsTroch_83292 | 3461 | -0.92 | 8.08E-03 | Inositol hexakisphosphate and  diphosphoinositol-pentakisphosphate kinase | *Phaseolus vulgaris* | 0.00E+00 |
| ScrippsTroch_90340 | 1739 | -0.91 | 3.86E-02 | Glucose-repressible alcohol dehydrogenase transcriptional effector | *Emiliania huxleyi* CCMP1516 | 3.11E-96 |
| ScrippsTroch_54924 | 3428 | -0.88 | 3.19E-02 | DNA excision repair protein ERCC-6 | *Sorghum bicolor* | 9.31E-111 |
| ScrippsTroch_55491 | 1868 | -0.86 | 4.80E-02 | UPF0187 protein sll1024 | *Perkinsus marinus* ATCC 50983 | 2.13E-23 |
| ScrippsTroch_36727 | 2954 | -0.82 | 2.91E-02 | Uncharacterized protein | *Emiliania huxleyi* CCMP1516 | 3.34E-145 |
| ScrippsTroch_16670 | 3586 | -0.70 | 3.16E-02 | ATP-dependent Clp protease ATP-binding subunit clpA homolog | *Prochlorococcus marinus* | 0.00E+00 |
| ScrippsTroch_97496 | 7771 | -0.70 | 1.95E-02 | Sushi, von Willebrand factor type A,  EGF and pentraxin domain-containing protein 1 | *Entamoeba invadens* IP1 | 1.77E-45 |
| ScrippsTroch_6005 | 4609 | -0.66 | 5.28E-02 | Multidrug resistance-associated protein 1 | *Trichechus manatus latirostris* | 2.77E-112 |
| ScrippsTroch_15963 | 3770 | 0.56 | 9.40E-02 | ATP-dependent Clp protease ATP-binding subunit clpA homolog | *Volvox carteri f. nagariensis* | 0.00E+00 |
| ScrippsTroch_10899 | 1517 | 0.78 | 3.54E-02 | 3CCCH domain containing protein | *Cryptosporidium parvum* Iowa II | 1.21E-14 |
| ScrippsTroch_82537 | 5370 | 0.89 | 1.15E-02 | Protein translocase subunit SecA 2 | *Polysphondylium pallidum* PN500 | 4.03E-75 |
| ScrippsTroch_7950 | 1552 | 0.98 | 1.99E-04 | cysteine proteinase | *Oxyrrhis marina* | 1.45E-66 |
| ScrippsTroch_54971 | 1967 | 1.06 | 1.76E-02 | UPF0187 protein sll1024 | *Perkinsus marinus* ATCC 50983 | 4.60E-26 |
| ScrippsTroch_17737 | 2726 | 1.11 | 6.72E-02 | Helicase carboxy-terminal domain protein, putative | *Reticulomyxa filosa* | 4.27E-18 |
| ScrippsTroch_15990 | 2723 | 1.15 | 3.09E-02 | Uncharacterized ABC transporter ATP-binding protein HI_0036 | *Polaromonas naphthalenivorans* CJ2 | 1.55E-81 |
| ScrippsTroch_17482 | 3576 | 1.28 | 2.09E-04 | Sushi, von Willebrand factor type A,  EGF and pentraxin domain-containing protein 1 | *Sinorhizobium fredii* USDA 257 | 9.09E-19 |
| ScrippsTroch_83755 | 3279 | 1.34 | 3.18E-03 | cathepsin B-like cysteine proteinase 3 | *Zea mays* | 1.52E-29 |
| ScrippsTroch_95807 | 1902 | 1.36 | 8.20E-03 | Cathepsin D | *Perkinsus marinus* ATCC 50983 | 2.98E-84 |
| ScrippsTroch_13816 | 1440 | 1.46 | 2.39E-11 | Zinc finger, CCCH type domain-containing protein | *Cryptosporidium muris* RN66 | 1.94E-09 |
| ScrippsTroch_69963 | 1140 | 1.48 | 7.99E-02 | ER lumen protein-retaining receptor A | *Cryptosporidium parvum* Iowa II | 2.21E-27 |
| ScrippsTroch_7050 | 1946 | 1.61 | 1.08E-03 | UPF0187 protein mll4386 | *Perkinsus marinus* ATCC 50983 | 1.19E-21 |
| ScrippsTroch_7435 | 1365 | 2.51 | 2.06E-05 | Zinc finger protein, putative | *Plasmodium knowlesi* strain H | 1.03E-12 |
| ScrippsTroch_9951 | 1493 | 2.66 | 6.64E-13 | Probable protein phosphatase 2C 34 | *Cryptosporidium muris* RN66 | 3.79E-48 |
| ScrippsTroch_83571 | 1242 | 3.53 | 1.50E-06 | Zinc finger protein, putative | *Plasmodium knowlesi* strain H | 5.07E-10 |
|  |  |  |  |  |  |  |
| **Signal Transduction** |  |  |  |  |  |  |
| Contig Name | Contig  Length | Log2  Fold Change | *P*-adjusted | Description | Best Blastx NR Hit Organism ID | Blast Hit  E-value |
| ScrippsTroch_16556 | 1870 | -2.00 | 1.10E-04 | Predicted protein | *Phaeodactylum tricornutum* CCAP 1055/1 | 1.86E-09 |
| ScrippsTroch_17457 | 4484 | -1.80 | 1.06E-21 | Protein NLRC3 | *Salpingoeca rosetta* | 1.10E-40 |
| ScrippsTroch_21432 | 1671 | -1.24 | 2.48E-04 | Vegetative incompatibility protein HET-E-1 | *Sclerotinia sclerotiorum* 1980 | 2.39E-50 |
| ScrippsTroch_88492 | 1536 | -1.19 | 7.21E-02 | CBL-interacting serine/threonine-protein kinase 18 | *Cucumis sativus* | 4.40E-22 |
| ScrippsTroch_74206 | 1880 | -1.04 | 6.32E-02 | Predicted protein | *Naegleria gruberi* | 1.79E-08 |
| ScrippsTroch_96140 | 1574 | -1.03 | 2.87E-04 | Blue-light-activated protein | *Aureococcus anophagefferens* | 1.12E-07 |
| ScrippsTroch_63537 | 4573 | -0.99 | 1.11E-05 | Uncharacterized protein | *Bathycoccus prasinos* | 0.00E+00 |
| ScrippsTroch_76066 | 1499 | -0.92 | 3.86E-02 | Protein NLRC3 | *Nematostella vectensis* | 1.05E-34 |
| ScrippsTroch_7576 | 3222 | -0.89 | 4.70E-02 | Sodium/calcium exchanger 3 | *Perkinsus marinus* ATCC 50983 | 6.93E-169 |
| ScrippsTroch_23342 | 1989 | -0.87 | 3.15E-06 | Uncharacterized protein | *Emiliania huxleyi* CCMP1516 | 4.66E-27 |
| ScrippsTroch_20285 | 2135 | -0.84 | 6.52E-02 | Vegetative incompatibility protein HET-E-1 | *Arthrospira platensi*s NIES-39 | 3.80E-31 |
| ScrippsTroch_23246 | 1249 | -0.84 | 4.72E-02 | Neurogenic locus notch homolog protein 2 | - | 8.21E-08 |
| ScrippsTroch_62847 | 1936 | -0.78 | 2.43E-02 | Zinc transporter ZIP12 | *Ostreococcus tauri* | 1.21E-45 |
| ScrippsTroch_48289 | 4949 | -0.75 | 3.31E-02 | Uncharacterized protein | *Bathycoccus prasinos* | 0.00E+00 |
| ScrippsTroch_66126 | 1912 | 0.85 | 6.64E-02 | Multi-drug resistance efflux pump PmrA | *Guillardia theta* CCMP2712 | 2.55E-41 |
| ScrippsTroch_20092 | 4680 | 0.87 | 7.49E-02 | Protein dispatched homolog 1 | *Chondrus crispus* | 2.56E-11 |
| ScrippsTroch_91768 | 2450 | 0.97 | 2.24E-02 | Protein MEI2-like 2 | *Perkinsus marinus* ATCC 50983 | 6.67E-29 |
| ScrippsTroch_88989 | 2783 | 1.15 | 2.03E-02 | SNF-related serine/threonine-protein kinase | *Oxytricha trifallax* | 6.64E-31 |
| ScrippsTroch_49291 | 1622 | 1.18 | 2.09E-02 | Myosin light chain kinase A | *Dictyostelium purpureum* | 1.91E-28 |
| ScrippsTroch_94035 | 3054 | 1.23 | 8.63E-03 | Calmodulin | *Prunus persica* | 1.23E-23 |
| ScrippsTroch_37958 | 2125 | 1.32 | 1.52E-03 | Putative uncharacterized protein | *Perkinsus marinus* ATCC 50983 | 4.68E-08 |
| ScrippsTroch_4902 | 1141 | 1.38 | 3.34E-02 | Probable protein S-acyltransferase 16 | *Perkinsus marinus* ATCC 50983 | 2.13E-79 |
| ScrippsTroch_55873 | 1363 | 1.71 | 1.22E-02 | Putative uncharacterized protein | Aureococcus anophagefferens | 6.00E-08 |
| ScrippsTroch_71332 | 1016 | 2.63 | 3.07E-02 | Uncharacterized protein | *Helobdella robusta* | 3.05E-06 |
|  |  |  |  |  |  |  |
| **Pathogen/Infection Related** | |  |  |  |  |  |
| Contig Name | Contig  Length | Log2  Fold Change | *P*-adjusted | Description | Best Blastx NR Hit Organism ID | Blast Hit  E-value |
| ScrippsTroch_89029 | 1880 | -2.47 | 3.07E-15 | Calcium-dependent protein kinase 3 | *Perkinsus marinus* ATCC 50983 | 1.88E-60 |
| ScrippsTroch_50784 | 735 | -1.97 | 1.41E-03 | RING-H2 finger protein ATL14 | *Physcomitrella patens* | 1.97E-07 |
| ScrippsTroch_659 | 1197 | -1.96 | 6.21E-09 | Concanavalin A-like lectin/glucanases family protein | Bacteriovorax sp. BSW11_IV | 6.20E-11 |
| ScrippsTroch_9569 | 1756 | -1.81 | 3.31E-06 | LIM and senescent cell antigen-like-containing domain protein 2 | *Cavia porcellus* | 1.31E-18 |
| ScrippsTroch_43634 | 1399 | 1.40 | 1.83E-04 | C-type lectin domain family 17, member A | *Takifugu rubripes* | 4.86E-12 |
| ScrippsTroch_11717 | 1129 | 1.65 | 1.46E-05 | hypothetical protein EXVG_00257 Emiliania huxleyi virus 202 | *Emiliania huxleyi* virus 202 | 5.19E-06 |
| ScrippsTroch_75684 | 857 | 1.90 | 7.14E-02 | Calcium-dependent protein kinase 28 | *Oryza sativa* Japonica Group | 6.81E-08 |
| ScrippsTroch_45823 | 1385 | 1.92 | 1.04E-03 | Calcium-dependent protein kinase 29 | *Paramecium tetraureli*a strain d4-2 | 2.65E-27 |
|  |  |  |  |  |  |  |
| **Ion Transporters, Metal Transporters** | | |  |  |  |  |
| Contig Name | Contig  Length | Log2  Fold Change | *P*-adjusted | Description | Best Blastx NR Hit Organism ID | Blast Hit  E-value |
| ScrippsTroch_1928 | 1070 | -1.83 | 4.17E-03 | Uncharacterized protein | *Plasmodium yoelii* 17X | 5.11E-10 |
| ScrippsTroch_64600 | 961 | -1.49 | 3.18E-03 | Uncharacterized protein | *Plasmodium yoelii* 17X | 3.09E-11 |
| ScrippsTroch_75031 | 2021 | -1.39 | 4.60E-02 | Uncharacterized protein | *Emiliania huxleyi* CCMP1516 | 5.34E-36 |
| ScrippsTroch_18588 | 1072 | -1.39 | 5.99E-03 | Plasma membrane iron permease | *Beauveria bassiana* ARSEF 2860 | 1.29E-09 |
| ScrippsTroch_55484 | 2332 | -1.28 | 2.84E-03 | Sodium channel protein type 11 subunit alpha | *Perkinsus marinus* ATCC 50983 | 4.95E-25 |
| ScrippsTroch_27816 | 1704 | -1.11 | 3.53E-02 | Abhydrolase domain-containing protein 1 | *Phaeodactylum tricornutum* CCAP 1055/1 | 9.35E-34 |
| ScrippsTroch_74707 | 4333 | -0.87 | 2.90E-02 | Probable sulfate transporter 3.3 | *Reinekea blandensis* | 9.51E-26 |
| ScrippsTroch_53224 | 3039 | -0.86 | 9.52E-02 | Putative uncharacterized protein | *Perkinsus marinus* ATCC 50983 | 1.46E-32 |
| ScrippsTroch_55128 | 2963 | -0.82 | 3.81E-02 | Putative uncharacterized protein | *Perkinsus marinus* ATCC 50983 | 1.93E-18 |
| ScrippsTroch_43797 | 2309 | -0.75 | 4.60E-02 | Sodium channel protein type 4 subunit alpha A | *Perkinsus marinus* ATCC 50983 | 8.12E-13 |
| ScrippsTroch_70590 | 3181 | -0.74 | 2.54E-02 | Putative uncharacterized protein | *Perkinsus marinus* ATCC 50983 | 5.78E-25 |
| ScrippsTroch_55794 | 3785 | -0.66 | 6.70E-02 | Sodium/hydrogen exchanger 8 | *Catenovulum agarivoran*s DS-2 | 2.01E-55 |
| ScrippsTroch_56888 | 9237 | -0.60 | 9.37E-02 | Potassium channel SKOR | *Nannochloropsis gaditana* | 7.98E-72 |
| ScrippsTroch_13016 | 7160 | -0.55 | 2.99E-02 | Hephaestin-like protein 1 | *Branchiostoma floridae* | 0.00E+00 |
| ScrippsTroch_33312 | 3596 | 0.74 | 5.45E-02 | Putative sulfate transporter YvdB | *Guillardia theta* CCMP2712 | 2.62E-104 |
| ScrippsTroch_4499 | 1800 | 1.05 | 2.27E-02 | Uncharacterized protein | *Emiliania huxleyi* CCMP1516 | 5.57E-31 |
| ScrippsTroch_37476 | 2334 | 1.47 | 3.48E-03 | Predicted protein | *Thalassiosira pseudonana* CCMP1335 | 5.59E-37 |
| ScrippsTroch_97190 | 1370 | 1.68 | 5.53E-05 | Uncharacterized protein | *Gregarina niphandrodes* | 4.58E-11 |
| ScrippsTroch_21283 | 1133 | 1.73 | 3.13E-02 | Solute carrier family 41 member 1 | *Branchiostoma floridae* | 1.62E-57 |
| ScrippsTroch_40340 | 928 | 2.34 | 6.77E-09 | Uncharacterized protein | *Plasmodium yoelii* 17X | 4.48E-14 |
|  |  |  |  |  |  |  |
| **Unknown** |  |  |  |  |  |  |
| Contig Name | Contig  Length | Log2  Fold Change | *P*-adjusted | Description | Best Blastx NR Hit Organism ID | Blast Hit  E-value |
| ScrippsTroch_1275 | 2268 | -2.35 | 1.55E-06 | Putative uncharacterized protein | *Pleurocapsa sp*. PCC 7319 | 9.99E-69 |
| ScrippsTroch_47405 | 1215 | -1.99 | 1.31E-06 | Putative uncharacterized protein | *Babesia microti* strain RI | 8.00E-06 |
| ScrippsTroch_11140 | 5219 | -1.80 | 7.38E-21 | Uncharacterized protein | *Pleurocapsa sp*. PCC 7319 | 4.77E-84 |
| ScrippsTroch_95461 | 2343 | -1.58 | 8.18E-07 | Putative uncharacterized protein | *Perkinsus marinus* ATCC 50983 | 1.06E-10 |
| ScrippsTroch_4093 | 1044 | -1.50 | 4.72E-03 | Uncharacterized protein | *Bathycoccus prasinos* | 1.13E-19 |
| ScrippsTroch_96214 | 2489 | -1.36 | 1.18E-08 | Repeat-containing protein A_01 | *Orientia tsutsugamushi str. Ikeda* | 1.69E-10 |
| ScrippsTroch_88296 | 3114 | -1.24 | 1.14E-04 | Putative ankyrin repeat protein L93 | *Strongylocentrotus purpuratus* | 9.82E-16 |
| ScrippsTroch_81583 | 4119 | -1.11 | 3.68E-02 | Putative uncharacterized protein | *Perkinsus marinus* ATCC 50983 | 4.28E-17 |
| ScrippsTroch_16874 | 3262 | -1.04 | 3.78E-03 | HEAT domain containing protein | *Trichodesmium erythraeum* IMS101 | 2.63E-27 |
| ScrippsTroch_101430 | 884 | -0.96 | 9.01E-02 | Uncharacterized protein | *Thalassiosira oceanica* | 2.89E-12 |
| ScrippsTroch_22611 | 5496 | -0.92 | 4.04E-06 | Uncharacterized protein | *Guillardia theta* CCMP2712 | 7.81E-109 |
| ScrippsTroch_23547 | 1835 | -0.90 | 1.16E-03 | Predicted protein | *Physcomitrella patens* | 1.14E-07 |
| ScrippsTroch_65842 | 2287 | -0.84 | 5.36E-02 | Uncharacterized protein | *Emiliania huxleyi* CCMP1516 | 1.29E-24 |
| ScrippsTroch_7680 | 1683 | -0.80 | 5.74E-02 | Uncharacterized protein | *Emiliania huxleyi* CCMP1516 | 0.00E+00 |
| ScrippsTroch_4079 | 2124 | -0.67 | 3.62E-02 | Uncharacterized protein | *Desulfovibrio magneticus* RS-1 | 2.82E-12 |
| ScrippsTroch_15514 | 3894 | -0.62 | 1.59E-02 | Uncharacterized protein | *Guillardia theta* CCMP2712 | 1.21E-90 |
| ScrippsTroch_33411 | 3749 | 0.83 | 1.59E-02 | Putative uncharacterized protein | *Perkinsus marinus* ATCC 50983 | 2.13E-47 |
| ScrippsTroch_107406 | 403 | 0.83 | 3.71E-04 | Putative uncharacterized protein | *Amphidinium carterae* | 7.31E-32 |
| ScrippsTroch_42323 | 2272 | 0.96 | 9.37E-02 | Uncharacterized protein | *Roseovarius sp*. 217 | 2.01E-54 |
| ScrippsTroch_21992 | 1186 | 0.99 | 9.57E-02 | Filamentous hemagglutinin family outer membrane protein | *Arcobacter nitrofigilis* DSM 7299 | 1.91E-32 |
| ScrippsTroch_54816 | 2835 | 1.07 | 2.03E-02 | Predicted protein | *Naegleria gruberi* | 2.00E-14 |
| ScrippsTroch_56466 | 3591 | 1.11 | 3.77E-03 | Uncharacterized protein | *Emiliania huxleyi* CCMP1516 | 2.65E-50 |
| ScrippsTroch_95800 | 1693 | 1.18 | 3.13E-02 | Uncharacterized protein | *Emiliania huxleyi* CCMP1516 | 5.73E-155 |
| ScrippsTroch_56068 | 1103 | 1.48 | 9.83E-02 | Putative uncharacterized protein | *Alexandrium fundyense* | 4.04E-06 |
| ScrippsTroch_16299 | 1752 | 1.63 | 3.79E-06 | Uncharacterized protein | *Emiliania huxleyi* CCMP1516 | 1.00E-71 |
| ScrippsTroch_88391 | 1447 | 1.75 | 9.25E-03 | Uncharacterized protein | *Oscillatoria nigro-vi*ridis PCC 7112 | 4.60E-10 |
| ScrippsTroch_40157 | 1209 | 1.80 | 5.95E-02 | Predicted protein | *Micromonas sp.* RCC299 | 4.25E-70 |
| ScrippsTroch_73754 | 1502 | 1.87 | 1.55E-03 | Transmembrane protein 43 | *Plesiocystis pacifica* | 1.98E-14 |
| ScrippsTroch_43928 | 903 | 2.67 | 2.82E-03 | Uncharacterized protein | *Emiliania huxleyi* CCMP1516 | 4.61E-61 |
| ScrippsTroch_54379 | 1297 | 2.67 | 1.63E-11 | Predicted protein | *Micromonas sp*. RCC299 | 3.66E-67 |

**Supplementary Literature Cited**

Armbrust, E.V., Berges, J.A., Bowler, C., Green, B.R., Martinez, D., Putnam, N.H., Zhou, S., Allen, A.E., Apt, K.E., and Bechner, M. (2004). The Genome of the Diatom *Thalassiosira pseudonana*: Ecology, Evolution, and Metabolism. *Science* 306**,** 79-86.

Bowler, C., Allen, A.E., Badger, J.H., Grimwood, J., Jabbari, K., Kuo, A., Maheswari, U., Martens, C., Maumus, F., Otillar, R.P., Rayko, E., Salamov, A., Vandepoele, K., Beszteri, B., Gruber, A., Heijde, M., Katinka, M., Mock, T., Valentin, K., Verret, F., Berges, J.A., Brownlee, C., Cadoret, J.-P., Chiovitti, A., Choi, C.J., Coesel, S., De Martino, A., Detter, J.C., Durkin, C., Falciatore, A., Fournet, J., Haruta, M., Huysman, M.J.J., Jenkins, B.D., Jiroutova, K., Jorgensen, R.E., Joubert, Y., Kaplan, A., Kroger, N., Kroth, P.G., La Roche, J., Lindquist, E., Lommer, M., Martin-Jezequel, V., Lopez, P.J., Lucas, S., Mangogna, M., Mcginnis, K., Medlin, L.K., Montsant, A., Secq, M.-P.O.-L., Napoli, C., Obornik, M., Parker, M.S., Petit, J.-L., Porcel, B.M., Poulsen, N., Robison, M., Rychlewski, L., Rynearson, T.A., Schmutz, J., Shapiro, H., Siaut, M., Stanley, M., Sussman, M.R., Taylor, A.R., Vardi, A., Von Dassow, P., Vyverman, W., Willis, A., Wyrwicz, L.S., Rokhsar, D.S., Weissenbach, J., Armbrust, E.V., Green, B.R., Van De Peer, Y., and Grigoriev, I.V. (2008). The *Phaeodactylum* genome reveals the evolutionary history of diatom genomes. *Nature* 456**,** 239-244.

Curtis, B.A., Tanifuji, G., Burki, F., Gruber, A., Irimia, M., Maruyama, S., Arias, M.C., Ball, S.G., Gile, G.H., Hirakawa, Y., Hopkins, J.F., Kuo, A., Rensing, S.A., Schmutz, J., Symeonidi, A., Elias, M., Eveleigh, R.J.M., Herman, E.K., Klute, M.J., Nakayama, T., Obornik, M., Reyes-Prieto, A., Armbrust, E.V., Aves, S.J., Beiko, R.G., Coutinho, P., Dacks, J.B., Durnford, D.G., Fast, N.M., Green, B.R., Grisdale, C.J., Hempel, F., Henrissat, B., Hoppner, M.P., Ishida, K.-I., Kim, E., Koreny, L., Kroth, P.G., Liu, Y., Malik, S.-B., Maier, U.G., Mcrose, D., Mock, T., Neilson, J.a.D., Onodera, N.T., Poole, A.M., Pritham, E.J., Richards, T.A., Rocap, G., Roy, S.W., Sarai, C., Schaack, S., Shirato, S., Slamovits, C.H., Spencer, D.F., Suzuki, S., Worden, A.Z., Zauner, S., Barry, K., Bell, C., Bharti, A.K., Crow, J.A., Grimwood, J., Kramer, R., Lindquist, E., Lucas, S., Salamov, A., Mcfadden, G.I., Lane, C.E., Keeling, P.J., Gray, M.W., Grigoriev, I.V., and Archibald, J.M. (2012). Algal genomes reveal evolutionary mosaicism and the fate of nucleomorphs. *Nature* 492**,** 59-65.

Fritz-Laylin, L.K., Prochnik, S.E., Ginger, M.L., Dacks, J.B., Carpenter, M.L., Field, M.C., Kuo, A., Paredez, A., Chapman, J., Pham, J., Shu, S., Neupane, R., Cipriano, M., Mancuso, J., Tu, H., Salamov, A., Lindquist, E., Shapiro, H., Lucas, S., Grigoriev, I.V., Cande, W.Z., Fulton, C., Rokhsar, D.S., and Dawson, S.C. (2010). The Genome of Naegleria gruberi Illuminates Early Eukaryotic Versatility. *Cell* 140**,** 631-642.

Gobler, C.J., Berry, D.L., Dyhrman, S.T., Wilhelm, S.W., Salamov, A., Lobanov, A.V., Zhang, Y., Collier, J.L., Wurch, L.L., Kustka, A.B., Dill, B.D., Shah, M., Verberkmoes, N.C., Kuo, A., Terry, A., Pangilinan, J., Lindquist, E.A., Lucas, S., Paulsen, I.T., Hattenrath-Lehmann, T.K., Talmage, S.C., Walker, E.A., Koch, F., Burson, A.M., Marcoval, M.A., Tang, Y.-Z., Lecleir, G.R., Coyne, K.J., Berg, G.M., Bertrand, E.M., Saito, M.A., Gladyshev, V.N., and Grigoriev, I.V. (2011). Niche of harmful alga Aureococcus anophagefferens revealed through ecogenomics. *Proceedings of the National Academy of Sciences* 108**,** 4352-4357.

King, N., Westbrook, M.J., Young, S.L., Kuo, A., Abedin, M., Chapman, J., Fairclough, S., Hellsten, U., Isogai, Y., Letunic, I., Marr, M., Pincus, D., Putnam, N., Rokas, A., Wright, K.J., Zuzow, R., Dirks, W., Good, M., Goodstein, D., Lemons, D., Li, W., Lyons, J.B., Morris, A., Nichols, S., Richter, D.J., Salamov, A., Sequencing, J.G.I., Bork, P., Lim, W.A., Manning, G., Miller, W.T., Mcginnis, W., Shapiro, H., Tjian, R., Grigoriev, I.V., and Rokhsar, D. (2008). The genome of the choanoflagellate Monosiga brevicollis and the origin of metazoans. *Nature* 451**,** 783-788.

Lamour, K.H., Mudge, J., Gobena, D., Hurtado-Gonzales, O.P., Schmutz, J., Kuo, A., Miller, N.A., Rice, B.J., Raffaele, S., Cano, L.M., Bharti, A.K., Donahoo, R.S., Finley, S., Huitema, E., Hulvey, J., Platt, D., Salamov, A., Savidor, A., Sharma, R., Stam, R., Storey, D., Thines, M., Win, J., Haas, B.J., Dinwiddie, D.L., Jenkins, J., Knight, J.R., Affourtit, J.P., Han, C.S., Chertkov, O., Lindquist, E.A., Detter, C., Grigoriev, I.V., Kamoun, S., and Kingsmore, S.F. (2012). Genome Sequencing and Mapping Reveal Loss of Heterozygosity as a Mechanism for Rapid Adaptation in the Vegetable Pathogen Phytophthora capsici. *Molecular Plant-Microbe Interactions* 25**,** 1350-1360.

Read, B.A., Kegel, J., Klute, M.J., Kuo, A., Lefebvre, S.C., Maumus, F., Mayer, C., Miller, J., Monier, A., Salamov, A., Young, J., Aguilar, M., Claverie, J.-M., Frickenhaus, S., Gonzalez, K., Herman, E.K., Lin, Y.-C., Napier, J., Ogata, H., Sarno, A.F., Shmutz, J., Schroeder, D., De Vargas, C., Verret, F., Von Dassow, P., Valentin, K., Van De Peer, Y., Wheeler, G., Emiliania Huxleyi Annotation, C., Dacks, J.B., Delwiche, C.F., Dyhrman, S.T., Glockner, G., John, U., Richards, T., Worden, A.Z., Zhang, X., and Grigoriev, I.V. (2013). Pan genome of the phytoplankton Emiliania underpins its global distribution. *Nature* 499**,** 209-213.

Sucgang, R., Kuo, A., Tian, X., Salerno, W., Parikh, A., Feasley, C.L., Dalin, E., Tu, H., Huang, E., Barry, K., Lindquist, E., Shapiro, H., Bruce, D., Schmutz, J., Salamov, A., Fey, P., Gaudet, P., Anjard, C., Babu, M.M., Basu, S., Bushmanova, Y., Wel, H., Katoh-Kurasawa, M., Dinh, C., Coutinho, P.M., Saito, T., Elias, M., Schaap, P., Kay, R.R., Henrissat, B., Eichinger, L., Rivero, F., Putnam, N.H., West, C.M., Loomis, W.F., Chisholm, R.L., Shaulsky, G., Strassmann, J.E., Queller, D.C., Kuspa, A., and Grigoriev, I.V. (2011). Comparative genomics of the social amoebae Dictyostelium discoideum and Dictyostelium purpureum. *Genome Biology* 12**,** 1-23.

Tyler, B.M., Tripathy, S., Zhang, X., Dehal, P., Jiang, R.H.Y., Aerts, A., Arredondo, F.D., Baxter, L., Bensasson, D., Beynon, J.L., Chapman, J., Damasceno, C.M.B., Dorrance, A.E., Dou, D., Dickerman, A.W., Dubchak, I.L., Garbelotto, M., Gijzen, M., Gordon, S.G., Govers, F., Grunwald, N.J., Huang, W., Ivors, K.L., Jones, R.W., Kamoun, S., Krampis, K., Lamour, K.H., Lee, M.-K., Mcdonald, W.H., Medina, M., Meijer, H.J.G., Nordberg, E.K., Maclean, D.J., Ospina-Giraldo, M.D., Morris, P.F., Phuntumart, V., Putnam, N.H., Rash, S., Rose, J.K.C., Sakihama, Y., Salamov, A.A., Savidor, A., Scheuring, C.F., Smith, B.M., Sobral, B.W.S., Terry, A., Torto-Alalibo, T.A., Win, J., Xu, Z., Zhang, H., Grigoriev, I.V., Rokhsar, D.S., and Boore, J.L. (2006). *Phytophthora* Genome Sequences Uncover Evolutionary Origins and Mechanisms of Pathogenesis. *Science* 313**,** 1261-1266.
